# Reduced fibroblast-derived IGFBP5 is associated with pathogenic synovial fibroblast states and altered macrophage polarization in rheumatoid arthritis

**DOI:** 10.3389/fimmu.2026.1873778

**Published:** 2026-08-26

**Authors:** Lunmin Bao, Daoming Wang, Ping Wang, Xiaoduo Li, Hailong Zhang, Xiufang Wan, Rui Yuan, Nanzi Xie, Mengting Shi, Shuchi Wu, Longfei Yue, Xian’e Cao, Haibing Dai, Ting Zheng, Hongmei Jiang

**Affiliations:** 1 Department of Immunology, School of Basic Medicine, Guizhou Medical University, Guiyang, Guizhou, China; 2 Department of Laboratory Medicine, People’s Hospital of Anshun City, Anshun, China; 3 Department of Clinical Microbiology and Immunology, School of Clinical Laboratory Science, Guizhou Medical University, Guiyang, China; 4 Department of Pathology, People’s Hospital of Anshun City, Anshun, China; 5 Department of General Practice, People’s Hospital of Anshun City, Anshun, China; 6 Department of Medical Technology, Zunyi Medical and Pharmaceutical College, Zunyi, China; 7 Department of Basic Medicine, Guizhou Nursing Vocational College, Guiyang, China

**Keywords:** IGFBP5, macrophage polarization, rheumatoid arthritis, scRNA-seq, synovial fibroblasts

## Abstract

**Background:**

Rheumatoid arthritis (RA) is characterized by synovial inflammation, synovial fibroblast (SF) activation, and joint destruction. Activated SFs acquire proliferative and invasive properties and interact with macrophages to maintain the inflammatory microenvironment. However, endogenous factors regulating fibroblast activation and fibroblast–macrophage communication in RA are not fully understood. In this study, we examined the expression of insulin-like growth factor binding protein 5 (IGFBP5) in RA synovium at single-cell resolution and investigated its role in SF activation and macrophage polarization.

**Methods:**

Single-cell RNA sequencing (scRNA-seq) datasets from normal and RA synovial tissues were reprocessed and integrated using Seurat and Harmony. Sample-level quality-control metrics and cellular composition were evaluated per sample. Donor-level pseudo-bulk analysis and external synovial transcriptomic datasets were used to validate IGFBP5 downregulation. Pseudotime analysis, SAVER imputation, RA-only CellChat analysis, and in silico IGFBP5 perturbation were performed. Disease-associated IGFBP5 expression was evaluated in a collagen-induced arthritis (CIA) mouse model, whereas IGFBP5 overexpression was examined *in vitro* using RA synovial fibroblasts and a Transwell fibroblast-macrophage coculture system.

**Results:**

scRNA-seq analysis showed that IGFBP5 expression was reduced in RA synovial fibroblasts, particularly in inflammatory and fibrotic subclusters. Donor-level pseudo-bulk analysis supported lower IGFBP5 expression in RA fibroblasts, particularly in lining and sublining fibroblasts, and external datasets showed concordant reductions or downward trends. IGFBP5 expression was negatively associated with pathogenic fibroblast markers FAP and THY1 and positively associated with the homeostatic marker PRG4. In CIA mice, IGFBP5 expression was decreased in hyperplastic synovial tissues and CIA-derived synovial fibroblasts. *In vitro*, IGFBP5 overexpression reduced fibroblast proliferation, migration, and invasion and increased apoptosis. In Transwell coculture assays, IGFBP5-overexpressing fibroblasts reduced CD86-associated and pro-inflammatory macrophage features and increased the CD86-negative/CD206-positive macrophage fraction and TGF-β and IL-10 secretion, whereas CD206 mean fluorescence intensity was not significantly changed.

**Conclusions:**

Reduced IGFBP5 expression is associated with pathogenic fibroblast states in RA. IGFBP5 overexpression *in vitro* attenuated aggressive features of RA synovial fibroblasts and altered macrophage phenotypic and cytokine profiles in coculture. These findings identify IGFBP5 as a fibroblast-associated pathway involved in synovial fibroblast-macrophage interactions and warrant *in vivo* intervention studies to establish causality and therapeutic efficacy.

## 1 Introduction

Rheumatoid arthritis (RA) is a chronic systemic autoimmune disease characterized by persistent synovial inflammation, progressive cartilage destruction, and bone erosion (1). A key pathological feature of RA is synovial lining hyperplasia and pannus formation, which contribute directly to joint damage. Synovial fibroblasts (SFs) are now recognized as active participants in this process rather than passive structural cells (2–4). In RA, activated SFs acquire aggressive features, including increased proliferation, resistance to apoptosis, and the ability to invade adjacent cartilage and bone (5, 6). Concurrently, they secrete cytokines and chemokines that recruit and polarize innate immune cells, particularly macrophages (7). Interactions between activated SFs and pro-inflammatory macrophages further amplify synovial inflammation and contribute to disease persistence (8). Therefore, identifying endogenous regulators that limit SF activation and fibroblast–macrophage crosstalk may provide new insight into RA pathogenesis.

Recent advances in single-cell RNA sequencing (scRNA-seq) have improved the characterization of cellular heterogeneity within the RA synovium (9, 10). Several fibroblast subsets with distinct pathological functions have been identified. For example, THY1^+^ and FAP^+^ sublining fibroblasts are associated with inflammation and tissue damage, whereas PRG4^+^ lining fibroblasts are linked to joint lubrication and synovial homeostasis (11, 12). However, the molecular factors associated with the transition from homeostatic fibroblast states to inflammatory or tissue-destructive phenotypes remain incompletely understood.

Insulin-like growth factor binding protein 5 (IGFBP5) is a secreted protein that regulates IGF signaling and also has IGF-independent effects on cell survival, migration, and extracellular matrix remodeling (13, 14). Altered IGFBP5 expression has been reported in fibrotic diseases and cancers, where its function appears to be context dependent (15–17). Despite these observations, the distribution of IGFBP5 across fibroblast subsets in RA synovium and its potential role in fibroblast activation and fibroblast–macrophage communication have not been fully investigated.

In this study, we combined single-cell transcriptomic analysis with *in vivo* assessment of disease-associated IGFBP5 expression and *in vitro* functional experiments to characterize IGFBP5 in RA. Using integrated scRNA-seq datasets of human synovial tissues, donor-level pseudo-bulk and external transcriptomic validation, pseudotime trajectory analysis, and in silico perturbation analysis, we found that IGFBP5 expression was reduced in pathogenic fibroblast subsets and declined along the inferred transition toward inflammatory and fibrotic fibroblast states. The disease-associated reduction of IGFBP5 was further evaluated in a collagen-induced arthritis (CIA) mouse model, whereas functional effects were examined using IGFBP5-overexpressing human RA synovial fibroblasts. We also used a Transwell coculture system to assess whether fibroblast-derived IGFBP5 alters macrophage phenotypic features. Our results suggest that reduced IGFBP5 is associated with a more pathogenic fibroblast state and may participate in fibroblast-macrophage interactions in RA.

## 2 Materials and methods

### 2.1 Collagen-induced arthritis mouse model

Male DBA/1 mice aged 8–10 weeks were maintained under specific pathogen-free conditions. Collagen-induced arthritis (CIA) was induced by intradermal injection of bovine type II collagen (CII, Chondrex) emulsified with complete Freund’s adjuvant at the base of the tail, followed by a booster injection of CII emulsified with incomplete Freund’s adjuvant on day 21. Normal control mice received saline. Five mice were included in each group. Arthritis severity was assessed by paw swelling and erythema. Hind-paw thickness and clinical arthritis scores were recorded longitudinally on days 14, 17, 21, 24, 27, 30, 33, and 36 after the initial immunization.

### 2.2 Cell culture

Primary synovial fibroblasts were isolated from joint synovial tissues of normal controls and CIA mice. Briefly, synovial tissues were minced, digested with 2 mg/mL collagenase type IV (C8160, Solarbio, Beijing, China) at 37 °C for 2 h, filtered, centrifuged, and cultured in DMEM supplemented with 10% fetal bovine serum and 1% penicillin/streptomycin. Non-adherent cells were removed after 24 h, and passages 3–6 were used for experiments.

The human RA synovial fibroblast cell line MH7A and THP-1 monocytic cell line were obtained from Procell (Wuhan, China). Primary fibroblasts and MH7A cells were cultured in DMEM containing 10% FBS and 1% penicillin/streptomycin. THP-1 cells were cultured in RPMI-1640 medium with the same supplements. All cells were maintained at 37 °C in a humidified incubator with 5% CO_2_.

### 2.3 Single-cell RNA sequencing analysis

scRNA-seq datasets of human synovial tissues from normal controls (NC; GSE246416, n = 5 samples) and RA patients (GSE200815, n = 4 samples) were downloaded from the Gene Expression Omnibus (https://www.ncbi.nlm.nih.gov/geo/). The Seurat R package (v5.0) was utilized for downstream bioinformatic processing (18). Cells were retained if they contained >300 and <5,000 detected genes, >1,000 unique molecular identifier (UMI) counts, <20% mitochondrial transcripts, and <3% hemoglobin-associated transcripts. To further exclude cells with unusually high transcript counts, cells with UMI counts above the 97th percentile within each sample were removed. Genes detected in fewer than three cells were excluded during Seurat object construction. Doublets were removed using DoubletFinder (19).

After normalization, the top 2,000 highly variable genes were selected. Batch effects were corrected using Harmony based on principal component analysis (20). UMAP was used for dimensionality reduction (21), and clustering was performed using FindNeighbors and FindClusters, with cluster resolution evaluated by Clustree (22). Cell types and fibroblast subclusters were annotated according to canonical marker genes and differentially expressed genes identified using the Wilcoxon rank-sum test.

### 2.4 Donor-level pseudo-bulk and external transcriptomic validation

To reduce cell-level pseudoreplication and assess the robustness of IGFBP5 downregulation at the sample level, donor-level pseudo-bulk analysis was performed. Raw RNA counts from cells belonging to the same donor were aggregated using AggregateExpression in Seurat. Because IGFBP5 expression was concentrated predominantly in fibroblast populations, pseudo-bulk validation focused on the overall fibroblast compartment and separately on lining, sublining, and proliferating fibroblasts. Differential expression between RA and NC samples was evaluated using DESeq2 with disease group as the design factor. DESeq2-normalized IGFBP5 counts were used for donor-level visualization.

IGFBP5 expression was further evaluated in external synovial transcriptomic datasets, including GSE55235, GSE55457, GSE77298, and GSE12021. Each dataset was processed and analyzed independently without cross-dataset normalization or merging. Expression values were log2 transformed when required, and RA-versus-control comparisons were performed using limma.

### 2.5 Imputation, correlation, trajectory, and cell–cell communication analyses

For fibroblast subsets, SAVER was used to reduce dropout effects in scRNA-seq data (23). Spearman correlation analysis and quadrant analysis were performed to assess the relationship between IGFBP5 and pathogenic fibroblast markers, including THY1, FAP, and CDH11, as well as the homeostatic marker PRG4.

Pseudotime trajectory analysis was performed using Monocle 3 (24). Harmony-corrected UMAP coordinates and clustering information from Seurat were imported into the CellDataSet object, and homeostatic fibroblast populations were defined as the trajectory root. Cell–cell communication analysis was conducted using CellChat to estimate interaction weights and ligand–receptor signaling patterns among cell populations (25). CellChat analysis was performed using pooled cells from the RA-only GSE200815 dataset (n = 4 donors; N = 27,699 cells) after quality-control filtering and doublet removal. The analyzed populations included fibroblasts, macrophages, T cells, B cells, endothelial cells, mural cells, and proliferating cells. No NC cells were included in this analysis. The RNA expression matrix and corresponding cell-type annotations were used to construct the CellChat object, and ligand–receptor interactions were inferred using the human CellChat database (CellChatDB.human). Communication probabilities were calculated using computeCommunProb, followed by pathway-level probability estimation and network aggregation. Interactions involving cell populations containing fewer than 10 cells were excluded. The CellChat analysis was intended to characterize predicted intercellular communication within the RA synovial environment rather than to perform a direct NC-versus-RA communication comparison.

### 2.6 *In silico* gene knockout and pathway enrichment analysis

Virtual IGFBP5 knockout was performed in homeostatic fibroblast subclusters using scTenifoldKnk (26). Gene expression perturbations after IGFBP5 deletion were calculated and compared with pathogenic fibroblast signatures. Differentially perturbed genes were analyzed by gene set enrichment analysis using clusterProfiler, including Gene Ontology, KEGG, and Reactome pathway analyses (27).

### 2.7 Histology and immunofluorescence staining

Mouse hind paws were fixed in 4% paraformaldehyde, decalcified in 10% EDTA, embedded in paraffin, and sectioned at 4 μm. H&E staining was performed to assess synovial hyperplasia, inflammatory infiltration, and cartilage/bone destruction. For tissue immunofluorescence, sections were incubated with antibodies against Vimentin (HA720165F, Huabio) and IGFBP5 (55205-1-AP, Proteintech), followed by fluorescent secondary antibodies.

For cellular immunofluorescence, cultured primary fibroblasts were fixed, permeabilized, and stained with antibodies against Vimentin, FAP (ET1704-23, Huabio), or IGFBP5. Nuclei were counterstained with DAPI, and images were acquired using a laser scanning confocal microscope (Nikon, Tokyo, Japan).

### 2.8 Lentiviral transduction for IGFBP5 overexpression

MH7A cells were transduced with lentiviral vectors encoding the full-length human IGFBP5 gene (OE group) or a negative control empty vector (NC group), synthesized by Genechem (Shanghai, China). Transduction was performed in the presence of polybrene (5 μg/mL). GFP expression was used to confirm transduction, and IGFBP5 overexpression was validated by Western blotting and ELISA. Wild-type (WT) MH7A cells were included as an additional control.

### 2.9 Cell proliferation, migration, invasion, apoptosis, and senescence assays

Cell viability was measured using CCK-8 assays (AC11L554, life-ilab, Shanghai, China) at 0, 24, 48, and 72 h. DNA synthesis and active proliferation were assessed using an EdU Cell Proliferation Kit (C0075S, Beyotime, Shanghai, China) according to the manufacturer’s protocol, with EdU-positive cells visualized and quantified under a fluorescence microscope. Cell migration was evaluated by wound-healing assay and Transwell migration assay. For invasion assays, Transwell chambers were pre-coated with Matrigel. After 24 h, migrated or invaded cells were fixed, stained with crystal violet, and counted.

Apoptosis was analyzed using Annexin V-FITC/PI staining (556547, BD Biosciences, USA) followed by flow cytometry (BD LSRFortessa™, BD Biosciences). Cellular senescence was assessed using SA-β-gal staining (C0602, Beyotime, China) according to the manufacturer’s instructions.

### 2.10 Macrophage differentiation and Transwell coculture system

THP-1 cells were differentiated into macrophages using phorbol 12-myristate 13-acetate (PMA, HY-18739, MCE, USA). To optimize differentiation, cells were treated with DMSO, 100 ng/mL PMA, or 200 ng/mL PMA, and differentiation efficiency was evaluated by morphology and CD11b expression. Based on these results, 100 ng/mL PMA was used for subsequent experiments.

For coculture assays, PMA-differentiated THP-1 macrophages were seeded in the lower chambers of 0.4-μm Transwell plates, while control or IGFBP5-overexpressing MH7A cells were seeded in the upper inserts. This system allowed soluble-factor-mediated communication without direct cell contact.

### 2.11 Flow cytometry analysis

Cells were harvested, washed with cold PBS, and incubated with Fc receptor blocking reagent before antibody staining. Flow-cytometry gating was performed sequentially by excluding debris on FSC-A/SSC-A plots, followed by singlet selection using FSC-A/FSC-H. Primary fibroblast purity was subsequently assessed using Vimentin and F4/80 staining. Apoptosis was evaluated using Annexin V-FITC/PI staining. For macrophage analyses, CD11b-positive macrophages were identified before assessment of CD86 and CD206 expression. Identical quadrant boundaries were applied to compared experimental groups. Representative four-quadrant analyses used for percentage quantification were displayed as contour plots where applicable. Data were acquired using a BD LSRFortessa flow cytometer and analyzed with FlowJo v10.10.0. Complete gating strategies are provided in the Supplementary Material.

### 2.12 Western blotting and enzyme-linked immunosorbent assay

Total intracellular proteins were extracted using RIPA lysis buffer supplemented with protease inhibitors. Equal amounts of proteins were separated by SDS-PAGE, transferred to PVDF membranes, and probed with primary antibodies against IGFBP5 and β-actin (internal control), followed by incubation with HRP-conjugated secondary antibodies. Protein bands were visualized using an enhanced chemiluminescence (ECL) detection system.

Conditioned culture supernatants from primary fibroblasts, MH7A cells, and macrophage cocultures were collected and centrifuged to remove cellular debris. Secreted levels of IGFBP5, TNF-α, IL-6, TGF-β, and IL-10 were quantified using specific human or mouse ELISA kits (ED-14530, ED-11776, ED-10377, ED-11801, ED-13626, LunChangShuoBiotech, China) according to the manufacturer’s instructions.

### 2.13 Statistical analysis

Statistical analyses were performed using GraphPad Prism (version 8.0.1) and R (version 4.3.0). Data are presented as mean ± SD or median with interquartile range, as indicated in individual figure legends. Two-group comparisons were performed using two-tailed Student’s t-tests or Wilcoxon rank-sum tests, as appropriate. Multiple-group comparisons were analyzed using one-way ANOVA followed by Tukey’s multiple-comparison test when applicable. Longitudinal paw-thickness and arthritis-score data were analyzed using two-way repeated-measures ANOVA with Geisser–Greenhouse correction and Šídák-adjusted multiple comparisons. Donor-level pseudo-bulk differential expression was analyzed using DESeq2, and external bulk transcriptomic datasets were analyzed using limma. Spearman correlation and Fisher’s exact tests were used for single-cell association analyses where indicated. The exact statistical test, sample size, and definition of replicates are specified in each figure legend. P < 0.05 was considered statistically significant (* P < 0.05; ** P < 0.01; *** P < 0.001; ns, not significant).

## 3 Results

### 3.1 Integration and annotation of human synovial single-cell datasets

**Figure 1:**
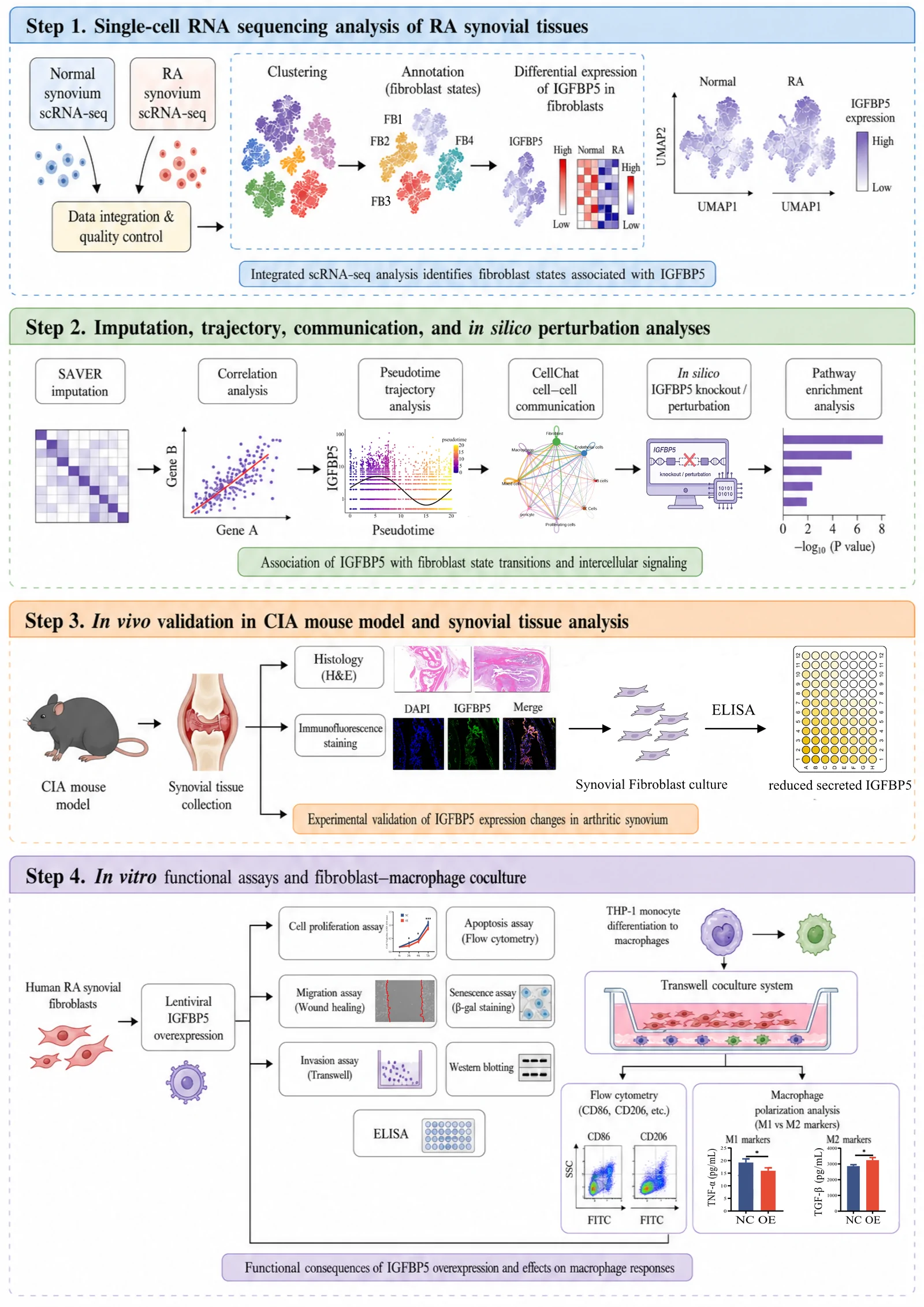
The flowchart of the overall study procedures.

The flow diagram of this study is illustrated in **Figure 1**. We analyzed scRNA-seq data from five NC samples (GSE246416) and four RA samples (GSE200815). Sample-level quality-control analyses showed that filtering removed cells with extreme UMI counts, detected gene numbers, and mitochondrial transcript proportions (**Supplementary Figures 1A–C**). The percentage of removed doublets was reported separately for each sample (**Supplementary Figure 1D**). Cellular composition and fibroblast abundance varied among individual samples and between datasets (**Supplementary Figure 2**).

Before integration, dataset-associated separation was evident in the UMAP representation. Harmony reduced this dataset-associated separation while preserving the major cellular structure (**Supplementary Figures 1E, F**).

**Figure 2:**
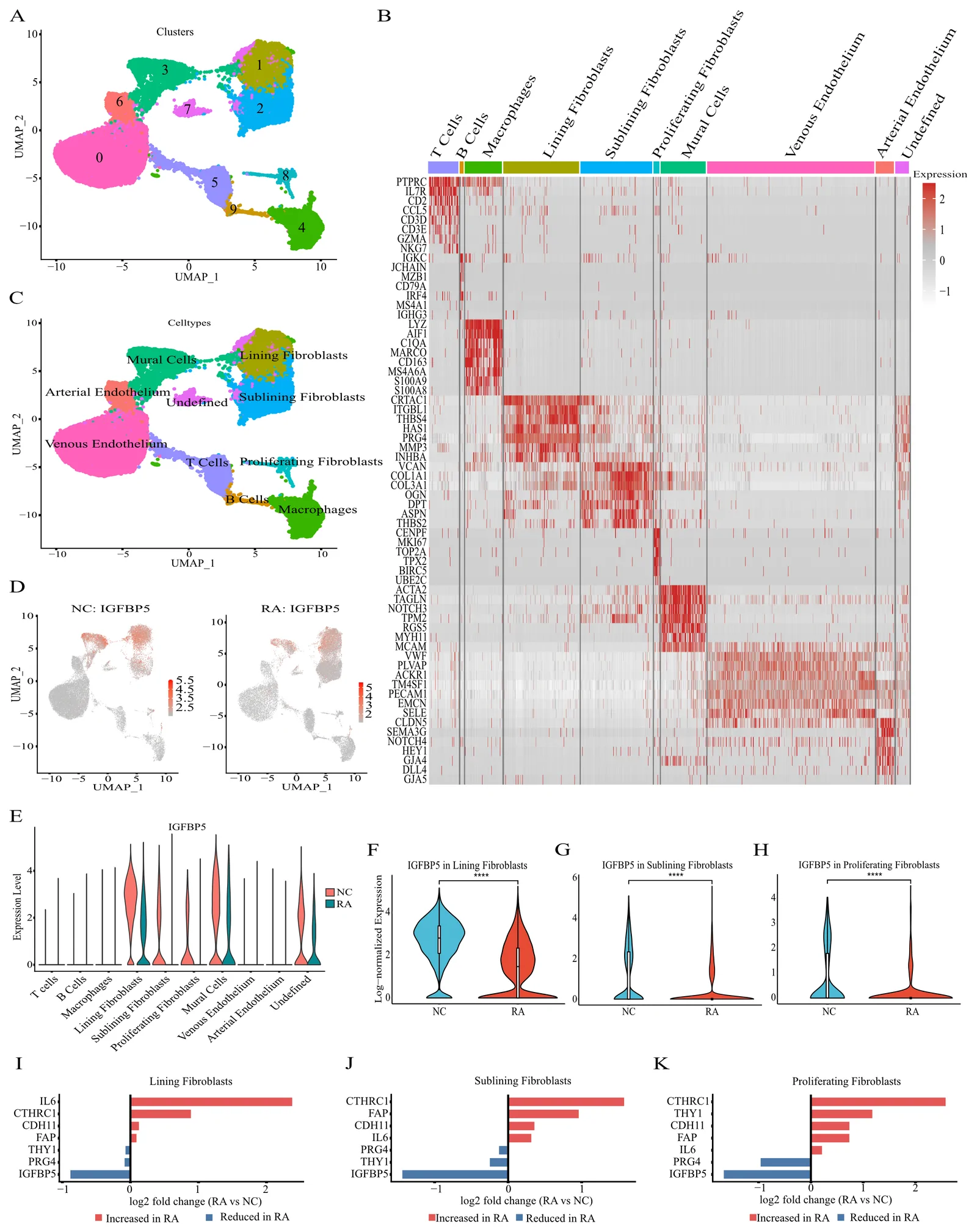
IGFBP5 expression is reduced in RA synovial fibroblasts. **(A)** UMAP plot of integrated scRNA-seq data from normal control (NC, n = 5; GSE246416) and RA synovial tissues (n = 4; GSE200815), showing 10 unsupervised clusters. **(B)** Heatmap of top marker genes across annotated cell types. Red indicates higher relative expression. **(C)** UMAP plot showing nine annotated cell populations together with an undefined population. **(D)** Feature plots showing IGFBP5 expression in NC and RA synovial tissues. IGFBP5 was mainly detected in fibroblast clusters and was reduced in RA. **(E)** Violin plots of IGFBP5 expression across annotated cell types in NC and RA groups. **(F–H)** Cell-level violin plots showing IGFBP5 expression in lining fibroblasts **(F)**, sublining fibroblasts **(G)**, and proliferating fibroblasts **(H)**. Cell-level comparisons were assessed using the Wilcoxon rank-sum test; ****P < 0.0001. **(I–K)** Horizontal bar plots showing relative RA-versus-NC log2 expression changes for IGFBP5, pathogenic fibroblast-associated markers (CDH11, THY1, CTHRC1, FAP, and IL6), and the homeostatic marker PRG4 in lining fibroblasts **(I)**, sublining fibroblasts **(J)**, and proliferating fibroblasts **(K)**.

Unsupervised clustering identified 10 cell clusters (**Figure 2A**), which were annotated into nine major cell populations based on canonical marker genes (**Figures 2B, C**). These included immune cells, including T cells, B cells, and macrophages, as well as stromal and vascular populations, including venous endothelial cells, arterial endothelial cells, mural cells, and synovial fibroblasts. Synovial fibroblasts were further divided into lining fibroblasts, sublining fibroblasts, and proliferating fibroblasts, marked by PRG4/MMP3, THY1/COL1A1, and MKI67/TOP2A, respectively.

### 3.2 IGFBP5 expression is reduced in RA synovial fibroblasts

We next examined the distribution of IGFBP5 across the annotated cell populations. Feature plots showed that IGFBP5 expression was mainly detected in fibroblast populations, with limited expression in immune or endothelial cells (**Figure 2D**). Compared with normal controls, RA synovial tissues showed reduced IGFBP5 expression, particularly within fibroblast populations (**Figure 2D**). Violin plots across major cell lineages further showed that the reduction was most evident in RA-derived synovial fibroblasts (**Figure 2E**). For descriptive context, pooled NC-versus-RA cell-type composition and IGFBP5 expression/positive-cell percentages across annotated populations are summarized in **Supplementary Figures 3A**, **S3B**, respectively.

### 3.3 IGFBP5 is reduced across fibroblast subclusters in RA

We further examined IGFBP5 expression within fibroblast subclusters. At the cell level, IGFBP5 transcript distributions were lower in RA-derived lining fibroblasts (**Figure 2F**, P < 0.0001), sublining fibroblasts (**Figure 2G**, P < 0.0001), and proliferating fibroblasts (**Figure 2H**, P < 0.0001) compared with the corresponding normal control subsets. Because individual cells are not independent biological replicates, these patterns were subsequently evaluated using donor-level pseudo-bulk analysis (Section 3.4).

We then compared IGFBP5 expression with markers associated with pathogenic or homeostatic fibroblast states, including CDH11, THY1, CTHRC1, FAP, IL6, and PRG4. Relative expression-change analysis showed that reduced IGFBP5 expression was accompanied by increased expression of several inflammatory or invasive fibroblast-associated markers, although the magnitude and direction of individual marker changes varied among fibroblast subsets (**Figures 2I–K**). In contrast, PRG4 expression showed a reduced or altered pattern in RA fibroblasts. These findings suggest that decreased IGFBP5 expression is associated with the shift of synovial fibroblasts toward inflammatory and tissue-destructive phenotypes in RA.

### 3.4 Donor-level and external transcriptomic validation of IGFBP5 downregulation

To evaluate whether the observed reduction of IGFBP5 was reproducible at the biological-sample level, we aggregated fibroblast raw counts within each donor and performed donor-level pseudo-bulk analysis. IGFBP5 expression was lower in the overall RA fibroblast compartment, with clear reductions in lining and sublining fibroblasts, whereas proliferating fibroblasts showed greater inter-donor variability (**Supplementary Figure 4A**).

We further evaluated IGFBP5 expression in multiple external synovial transcriptomic datasets. Lower IGFBP5 expression in RA was reproduced in several datasets, including GSE55235, GSE55457, and GSE12021, whereas GSE77298 showed a weaker downward pattern (**Supplementary Figure 4B**). The magnitude of the difference varied among datasets. Together, these complementary analyses support the reproducibility of reduced IGFBP5 expression while acknowledging that residual heterogeneity between the original scRNA-seq datasets cannot be completely excluded.

### 3.5 IGFBP5 expression is enriched in PRG4-positive fibroblasts in RA synovium

**Figure 3:**
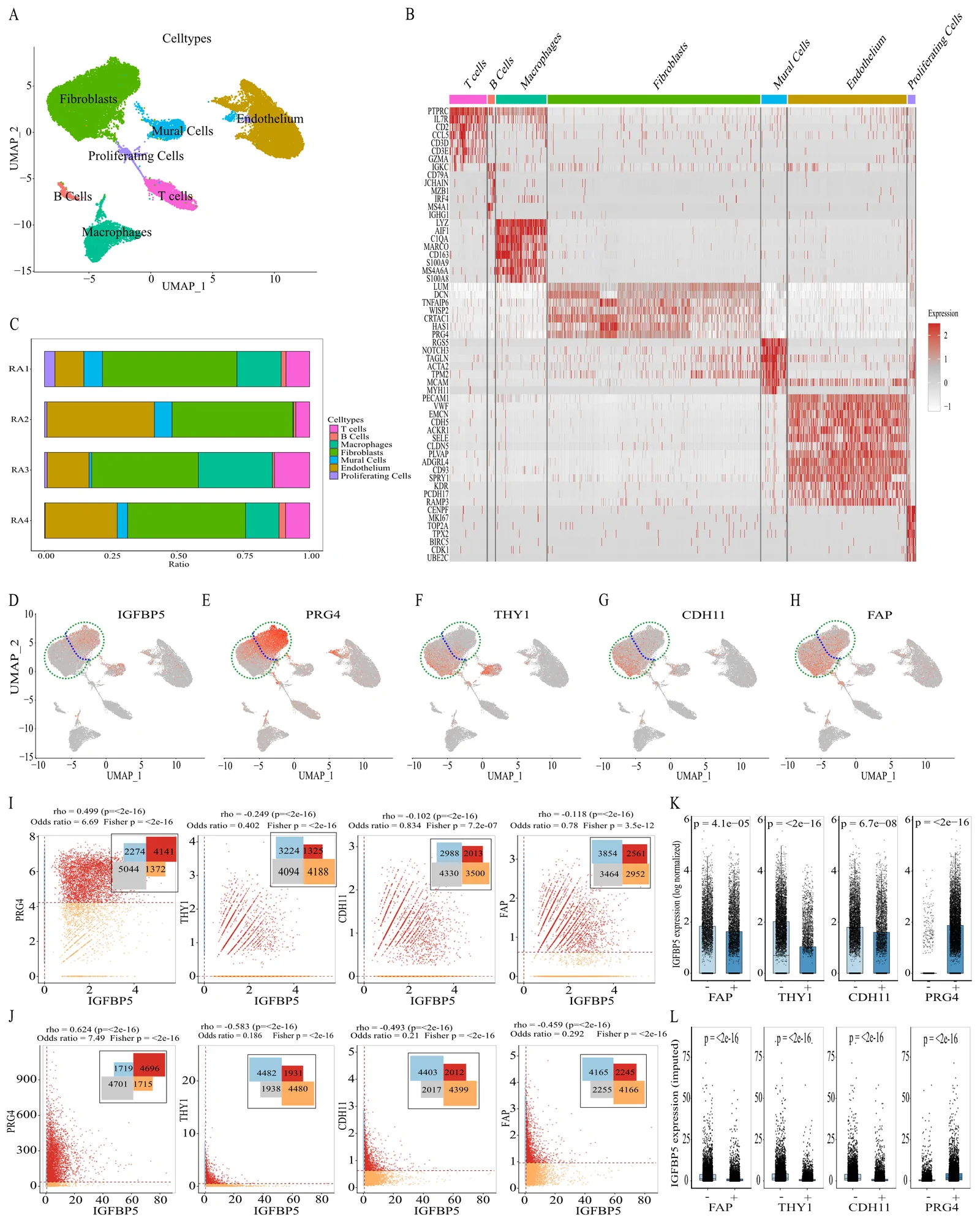
IGFBP5 expression is associated with PRG4-positive fibroblasts and inversely associated with pathogenic fibroblast markers in RA synovium. **(A)** UMAP plot of major cell populations in the RA synovial dataset (GSE200815, n = 4). **(B)** Heatmap of top marker genes used for cell type annotation. **(C)** Stacked bar plots showing the relative proportions of cell populations in four RA samples. **(D–H)** Feature plots showing the expression of IGFBP5 **(D)**, PRG4 **(E)**, THY1 **(F)**, CDH11 **(G)**, and FAP **(H)**. The green dashed line indicates the fibroblast compartment, and the blue dashed line marks a clear boundary between a region enriched for IGFBP5 and PRG4 expression and regions with higher THY1, CDH11, and FAP expression. **(I, J)** Scatter plots and quadrant analyses showing the associations between IGFBP5 and PRG4, THY1, CDH11, or FAP in RA fibroblasts using log-normalized data **(I)** and SAVER-imputed data **(J)**. Spearman’s correlation coefficient, Fisher’s exact test P-values, and odds ratios are shown. **(K, L)** Box and dot plots comparing IGFBP5 expression between marker-positive and marker-negative fibroblasts for FAP, THY1, CDH11, and PRG4 using log-normalized data **(K)** and SAVER-imputed data **(L)**. Statistical significance was assessed using the Wilcoxon rank-sum test.

To examine IGFBP5-expressing cells within RA synovium, we analyzed the RA dataset separately (GSE200815, n = 4). UMAP clustering identified the major cell populations, including fibroblasts, macrophages, T cells, B cells, endothelial cells, mural cells, and proliferating cells (**Figure 3A**). Cluster identities were assigned according to canonical marker genes (**Figure 3B**). The relative proportions of immune and stromal cell populations varied among individual RA samples, and fibroblasts accounted for the highest proportion in the synovium of RA patients (**Figure 3C**).

We then mapped IGFBP5 and selected fibroblast markers onto the fibroblast compartment (**Figures 3D–H**). IGFBP5 expression overlapped mainly with PRG4-positive fibroblasts (**Figures 3D, E**). In contrast, regions with higher expression of THY1, CDH11, and FAP showed lower IGFBP5 expression (**Figures 3F–H**), suggesting that IGFBP5 is preferentially expressed in fibroblasts with a more homeostatic marker profile.

### 3.6 IGFBP5 is inversely associated with pathogenic fibroblast markers

To further evaluate these expression patterns, we performed single-cell correlation and quadrant analyses in RA fibroblasts. In log-normalized data, IGFBP5 showed a positive correlation with PRG4 (Spearman’s rho = 0.499, odds ratio = 6.69, P < 2e-16) and negative correlations with THY1, CDH11, and FAP (**Figure 3I**). Similar patterns were observed after SAVER imputation, with clearer inverse associations between IGFBP5 and the pathogenic fibroblast markers THY1, CDH11, and FAP (**Figure 3J**).

We next compared IGFBP5 expression between marker-positive and marker-negative fibroblast subsets. IGFBP5 expression was lower in FAP^+^, THY1^+^, and CDH11^+^ fibroblasts than in their corresponding marker-negative subsets (P < 4.1e-05, P < 2e-16, and P = 6.7e-08, respectively), whereas PRG4^+^ fibroblasts showed higher IGFBP5 expression (**Figure 3K**). These trends were also observed in the imputed dataset (**Figure 3L**). Together, these results indicate that IGFBP5 expression is associated with PRG4-positive fibroblasts and is reduced in fibroblast subsets expressing pathogenic markers in RA synovium.

### 3.7 IGFBP5 is reduced in inflammatory and fibrotic fibroblast subsets

**Figure 4:**
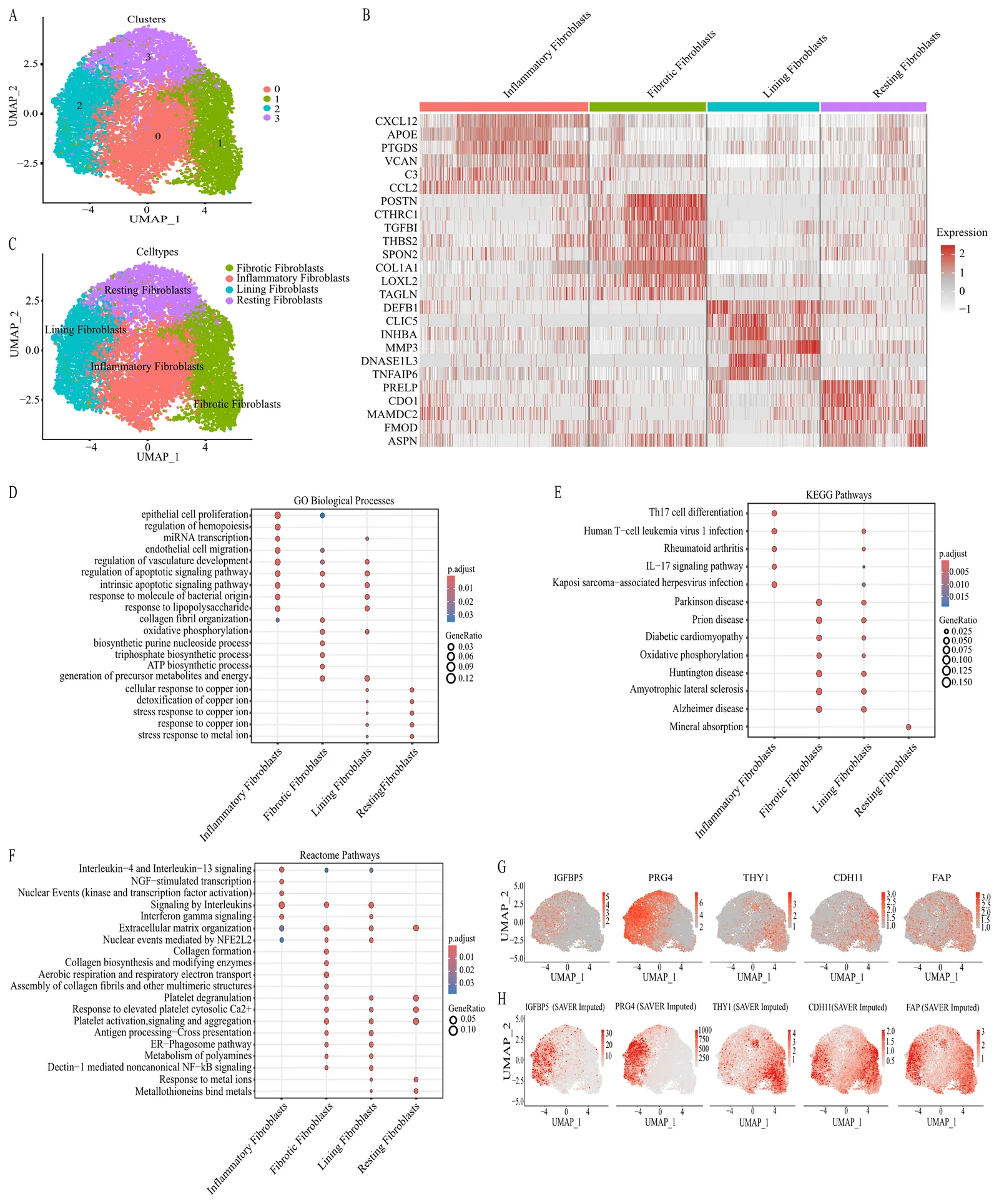
IGFBP5 expression is reduced in inflammatory and fibrotic fibroblast subsets in RA. **(A)** UMAP plot of reclustered synovial fibroblasts from the RA dataset, showing four fibroblast subsets. **(B)** Heatmap of top marker genes for inflammatory, fibrotic, lining, and resting fibroblasts. **(C)** UMAP plot showing the distribution and annotation of the four fibroblast subsets. **(D–F)** Functional enrichment analysis of the fibroblast subsets based on Gene Ontology biological processes **(D)**, KEGG pathways **(E)**, and Reactome pathways **(F)**. **(G, H)** Feature plots showing IGFBP5, PRG4, THY1, CDH11, and FAP expression across fibroblast subsets using log-normalized data **(G)** and SAVER-imputed data **(H)**.

As shown in **Figures 3D–H**, a clear boundary was observed within the fibroblast compartment, with IGFBP5/PRG4-enriched regions separated from areas expressing high levels of THY1, CDH11, and FAP. This prompted us to perform subcluster analysis of fibroblasts. Unsupervised analysis identified four fibroblast subsets (**Figure 4A**), which were annotated according to their marker genes (**Figures 4B, C**): inflammatory fibroblasts (CXCL12, CCL2, and C3), fibrotic fibroblasts (POSTN, CTHRC1, and COL1A1), lining fibroblasts (MMP3 and TNFAIP6), and resting fibroblasts (PRELP and ASPN).

To examine the functional features of these subsets, we performed enrichment analyses using GO, KEGG, and Reactome databases. Inflammatory fibroblasts were enriched in immune-related and inflammatory pathways, including response to lipopolysaccharide, IL-17 signaling, and interleukin-4/13 signaling, whereas fibrotic fibroblasts were enriched in extracellular matrix-related pathways, including collagen fibril organization, extracellular matrix organization, and collagen biosynthesis (**Figures 4D–F**).

We then mapped IGFBP5 expression across these fibroblast subsets. In both original and imputed data, THY1, CDH11, and FAP were mainly expressed in inflammatory and fibrotic fibroblasts. In contrast, IGFBP5 and PRG4 expression was lower in these two subsets and relatively higher in resting and lining fibroblasts (**Figures 4G, H**). These results indicate that reduced IGFBP5 expression is associated with inflammatory and fibrotic fibroblast states in RA.

Among RA fibroblasts, inflammatory, fibrotic, lining, and resting fibroblasts accounted for 33.5%, 23.2%, 22.3%, and 21.0% of cells, respectively (**Supplementary Figure 5A**). The proportion of IGFBP5-positive cells was highest in lining fibroblasts (79.9%), followed by resting (44.2%), inflammatory (36.0%), and fibrotic fibroblasts (17.0%) (**Supplementary Figure 5B**). Consistently, normalized IGFBP5 expression was higher in lining and resting fibroblasts than in inflammatory and fibrotic fibroblasts (**Supplementary Figure 5C**).

### 3.8 IGFBP5 expression decreases along the inferred fibroblast trajectory

To explore the relationship between IGFBP5 expression and fibroblast state changes, we performed pseudotime trajectory analysis. The inferred trajectory started mainly from resting and lining fibroblasts and extended toward inflammatory and fibrotic fibroblasts at later pseudotime points (**Supplementary Figure 6A**).

IGFBP5 expression was higher near the early pseudotime region and gradually decreased along branches leading to inflammatory and fibrotic fibroblast subsets (**Supplementary Figure 6B**). Gene expression modeling showed a similar pattern, with higher IGFBP5 levels in early pseudotime and lower expression at later stages (**Supplementary Figures 6C, D**). These results suggest that reduced IGFBP5 expression is associated with the inferred transition from resting/lining fibroblast states toward inflammatory and fibrotic states in RA.

### 3.9 IGFBP5 expression is reduced in synovial fibroblasts of CIA mice

**Figure 5:**
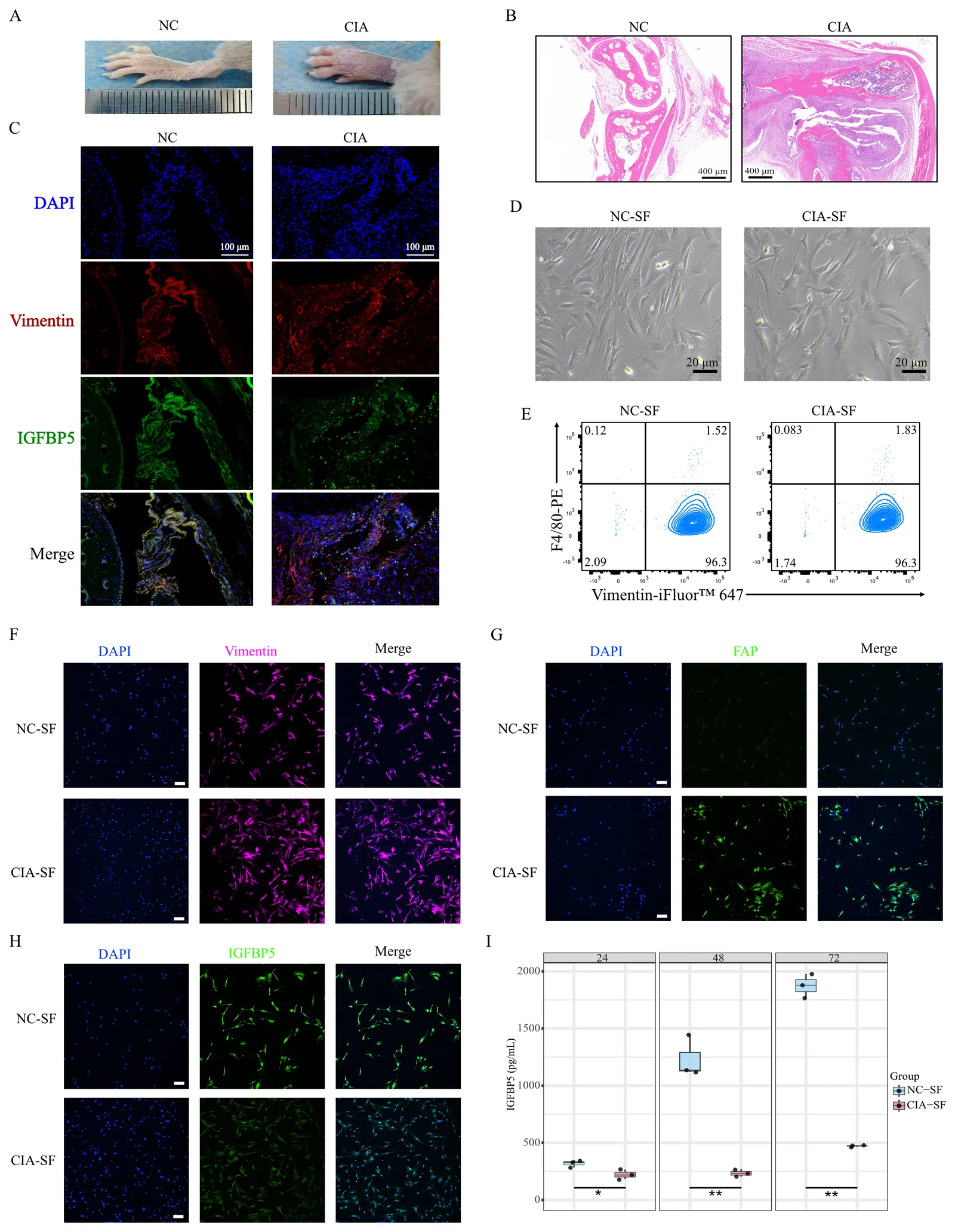
IGFBP5 expression is reduced in synovial fibroblasts from CIA mice. **(A)** Representative hind-paw images from normal control (NC) and collagen-induced arthritis (CIA) mice. **(B)** H&E staining of joint sections from NC and CIA mice. Scale bars = 400 μm. **(C)** Co-immunofluorescence staining of joint sections for IGFBP5 (green) and Vimentin (red); nuclei were stained with DAPI (blue). Scale bars = 100 μm. **(D)** Bright-field images of primary synovial fibroblasts isolated from NC and CIA mice. Scale bars = 20 μm. **(E)** Flow-cytometry assessment of primary fibroblast purity using Vimentin and F4/80. **(F–H)** Immunofluorescence staining of cultured NC-SF and CIA-SF for Vimentin **(F)**, FAP **(G)**, and IGFBP5 **(H)**; nuclei were stained with DAPI. Scale bars = 100 μm. **(I)** ELISA measurement of secreted IGFBP5 in NC-SF and CIA-SF culture supernatants at 24, 48, and 72 h. Time-course comparisons were analyzed using two-way ANOVA followed by Šídák’s multiple-comparisons test (n = 3). *P < 0.05, **P < 0.01.

Given the limited availability of healthy human synovial tissues, we established a collagen-induced arthritis (CIA) mouse model to determine whether the disease-associated reduction of IGFBP5 observed in human scRNA-seq data was also present in experimental arthritis. Compared with normal control mice, CIA mice developed paw swelling and erythema (**Figure 5A**). H&E staining showed synovial hyperplasia, inflammatory cell infiltration, and cartilage/bone erosion in CIA joints, whereas these changes were not observed in normal joints (**Figure 5B**). Longitudinal assessment further showed progressive increases in hind-paw thickness and arthritis scores in CIA mice compared with NC mice (**Supplementary Figures 7A, B**).

We next performed co-immunofluorescence staining for IGFBP5 and the fibroblast marker Vimentin in joint sections. In normal mice, IGFBP5 was detected in Vimentin-positive synovial fibroblasts in the lining region. In CIA mice, IGFBP5 staining was reduced in the expanded Vimentin-positive synovial fibroblast population (**Figure 5C**). These results are consistent with the scRNA-seq findings and indicate reduced IGFBP5 expression in synovial fibroblasts during arthritis.

### 3.10 Primary CIA-derived synovial fibroblasts show reduced IGFBP5 expression and secretion

We then isolated primary synovial fibroblasts from normal control and CIA mice. Both NC-SF and CIA-SF showed typical spindle-shaped fibroblast morphology (**Figure 5D**). Flow cytometry showed that more than 96% of the isolated cells were Vimentin-positive and F4/80-negative, supporting their fibroblast identity and low macrophage contamination (**Figure 5E**). The complete gating strategy is shown in **Supplementary Figure 8A**.

Immunofluorescence staining showed Vimentin expression in both NC-SF and CIA-SF (**Figure 5F**). Compared with NC-SF, CIA-SF showed higher FAP expression and lower intracellular IGFBP5 expression (**Figures 5G, H**). Because IGFBP5 is a secreted protein, we also measured IGFBP5 levels in culture supernatants. ELISA showed that secreted IGFBP5 increased over time in NC-SF cultures, whereas CIA-SF produced lower levels of IGFBP5 at 24, 48, and 72 h (P < 0.05 at 24 h; P < 0.01 at 48 and 72 h; **Figure 5I**). These data suggest that reduced intracellular and secreted IGFBP5 is associated with the activated phenotype of CIA-derived synovial fibroblasts.

### 3.11 CellChat and in silico perturbation analyses suggest potential roles of IGFBP5 in fibroblast activation and macrophage crosstalk

**Figure 6:**
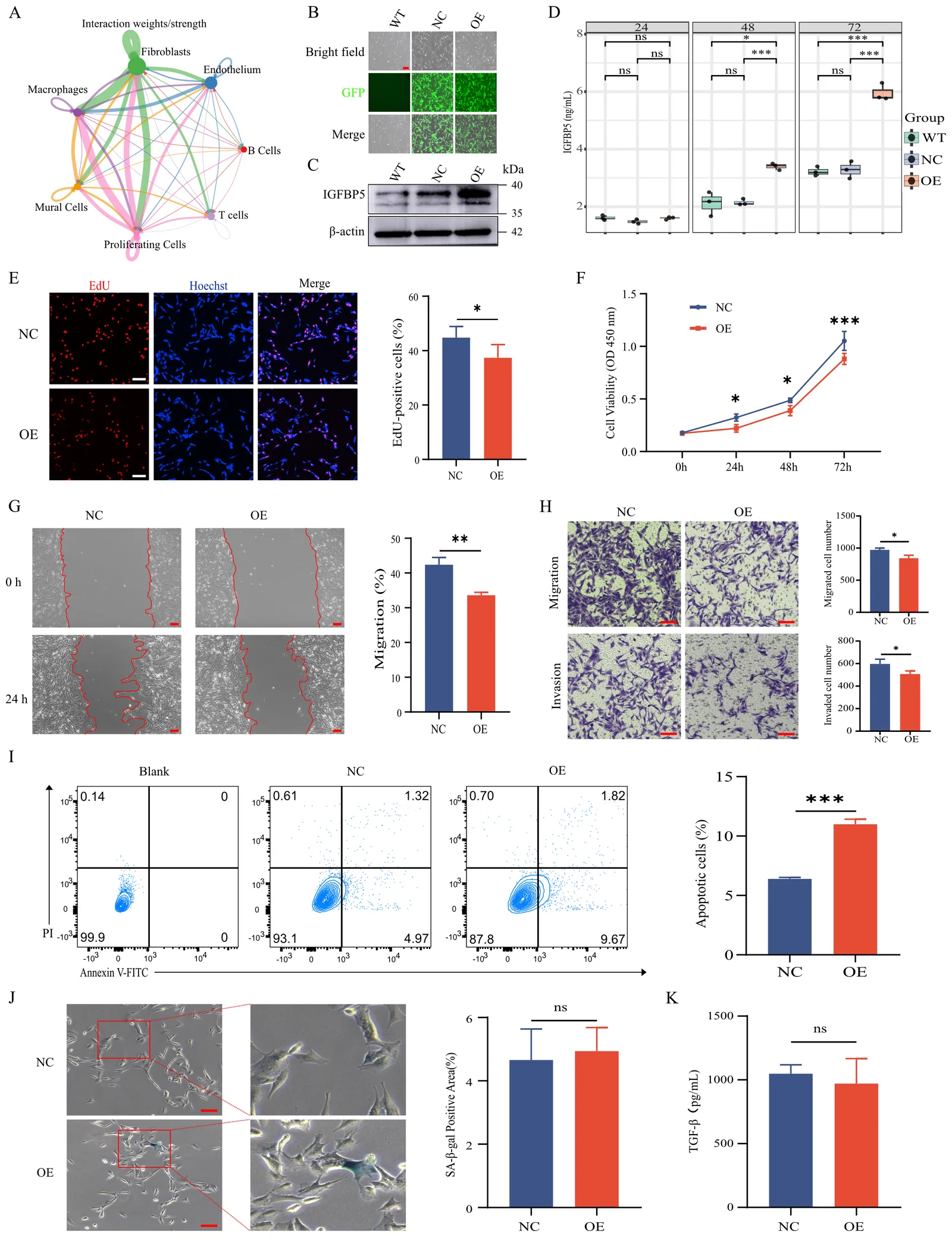
IGFBP5 overexpression reduces proliferation, migration, and invasion of RA synovial fibroblasts *in vitro*. **(A)** CellChat analysis of predicted aggregate interaction weights among major cell populations in the RA-only GSE200815 dataset (n = 4 donors; N = 27,699 cells). Fibroblasts showed prominent interactions with themselves and with macrophages. **(B)** Representative fluorescence images of MH7A cells after lentiviral transduction. GFP-positive cells indicate successful transduction (OE, IGFBP5 overexpression; NC, empty-vector control). Scale bars = 60 μm. **(C)** Representative Western blot showing IGFBP5 protein expression in WT, NC, and OE MH7A cells. **(D)** ELISA measurement of secreted IGFBP5 in the culture supernatants of WT, NC, and OE MH7A cells at 24, 48, and 72 h (n = 3). **(E)** EdU assay showing DNA synthesis in MH7A cells. Red indicates EdU-positive cells and blue indicates DAPI-stained nuclei. The percentage of EdU-positive cells was quantified (n = 5). Scale bars = 100 μm. **(F)** CCK-8 assay showing cell viability in WT, NC, and OE MH7A cells over 72 h (n = 4). **(G)** Wound-healing assay of MH7A cells, with migration quantified as the percentage of wound closure at 24 h (n = 3). Scale bars = 50 μm. **(H)** Transwell migration and invasion assays of MH7A cells (n = 3). Representative images and quantification of migrated and invaded cells are shown. Scale bars = 50 μm. **(I)** Flow cytometry analysis of apoptosis using Annexin V-FITC/PI staining. The percentage of apoptotic cells was quantified (n = 3). **(J)** SA-β-gal staining of MH7A cells. No significant difference was observed between NC and OE groups (n = 4). Scale bars = 50 μm. **(K)** ELISA measurement of TGF-β levels in culture supernatants from NC and OE groups. No significant difference was observed (n = 3). Time-course data were analyzed using two-way ANOVA followed by Šídák’s multiple-comparisons test; two-group endpoint comparisons were analyzed using two-tailed Student’s t-tests, and three-group endpoint comparisons were analyzed using one-way ANOVA followed by Tukey’s multiple-comparisons test. *P < 0.05, **P < 0.01, ***P < 0.001; ns, not significant.

To explore potential fibroblast-mediated interactions in the RA synovium, we performed CellChat analysis using the RA-only GSE200815 dataset. Fibroblasts showed high outgoing and incoming interaction weights, suggesting that they may act as an important signaling population in the RA synovial microenvironment (**Figure 6A**; **Supplementary Figures 9A, B**). The predicted interaction network indicated both fibroblast–fibroblast communication and interactions between fibroblasts and immune cells, particularly macrophages (**Figure 6A**).

We next performed in silico IGFBP5 knockout in resting and lining fibroblast subsets to explore its potential functional effects. Virtual IGFBP5 deletion induced predicted transcriptomic changes in these fibroblast populations (**Supplementary Figure 10A**). Signature shift analysis suggested that IGFBP5 loss was associated with a shift toward inflammatory and fibrotic fibroblast states (**Supplementary Figure 10B**).

GSEA further showed that virtual IGFBP5 knockout in lining fibroblasts was associated with reduced enrichment of “negative regulation of growth” and increased enrichment of immune-related pathways, including T cell- and B cell-mediated immunity (**Supplementary Figure 10C**). In resting fibroblasts, IGFBP5 perturbation was associated with pathways related to tissue remodeling and immune activation, including “response to wounding” and “antigen processing and presentation” (**Supplementary Figure 10D**). Based on these predictions, we next examined whether IGFBP5 affects fibroblast proliferation, migration, invasion, and macrophage phenotypic responses *in vitro*.

### 3.12 IGFBP5 overexpression reduces proliferation, migration, and invasion of RA synovial fibroblasts

To assess the functional role of IGFBP5 in RA synovial fibroblasts, we established IGFBP5-overexpressing MH7A cells, together with wild-type and empty-vector control cells. Lentiviral transduction was confirmed by GFP expression (**Figure 6B**). Western blotting and ELISA showed increased intracellular and secreted IGFBP5 levels in the overexpression group over 72 h (**Figures 6C, D**).

We then examined the effect of IGFBP5 on fibroblast proliferation. EdU staining showed that IGFBP5 overexpression reduced the proportion of EdU-positive cells (P < 0.05; **Figure 6E**). Consistently, CCK-8 assays showed lower cell viability in IGFBP5-overexpressing cells compared with control cells over time (**Figure 6F**). Wound-healing and Transwell assays further showed that IGFBP5 overexpression reduced the migratory and invasive abilities of MH7A cells (**Figures 6G, H**).

We next assessed whether IGFBP5 affected apoptosis or senescence. Flow cytometry showed an increased proportion of apoptotic cells in the IGFBP5-overexpressing group (**Figure 6I**); the complete gating strategy is shown in **Supplementary Figure 8B**. In contrast, IGFBP5 overexpression did not significantly change SA-β-gal staining (**Figure 6J**) or TGF-β secretion (**Figure 6K**), and TNF-α and IL-6 were not detectable in the culture supernatants. These results suggest that IGFBP5 overexpression reduces several aggressive features of RA synovial fibroblasts and promotes apoptosis without inducing an evident senescence phenotype.

### 3.13 THP-1 macrophage differentiation for coculture experiments

To examine the effect of fibroblast-derived IGFBP5 on macrophages, THP-1 monocytes were differentiated with PMA. We compared DMSO, 100 ng/mL PMA, and 200 ng/mL PMA to determine the working concentration. Cells treated with 100 ng/mL PMA showed adherent macrophage-like morphology (**Supplementary Figure 11A**) and higher CD11b expression than the DMSO control (**Supplementary Figure 11B**). Baseline analysis of CD86 and CD206 expression supported the use of 100 ng/mL PMA for macrophage differentiation (**Supplementary Figure 11C**). Therefore, 100 ng/mL PMA was used in subsequent coculture experiments.

### 3.14 Fibroblast-derived IGFBP5 modulates macrophage polarization in coculture

**Figure 7:**
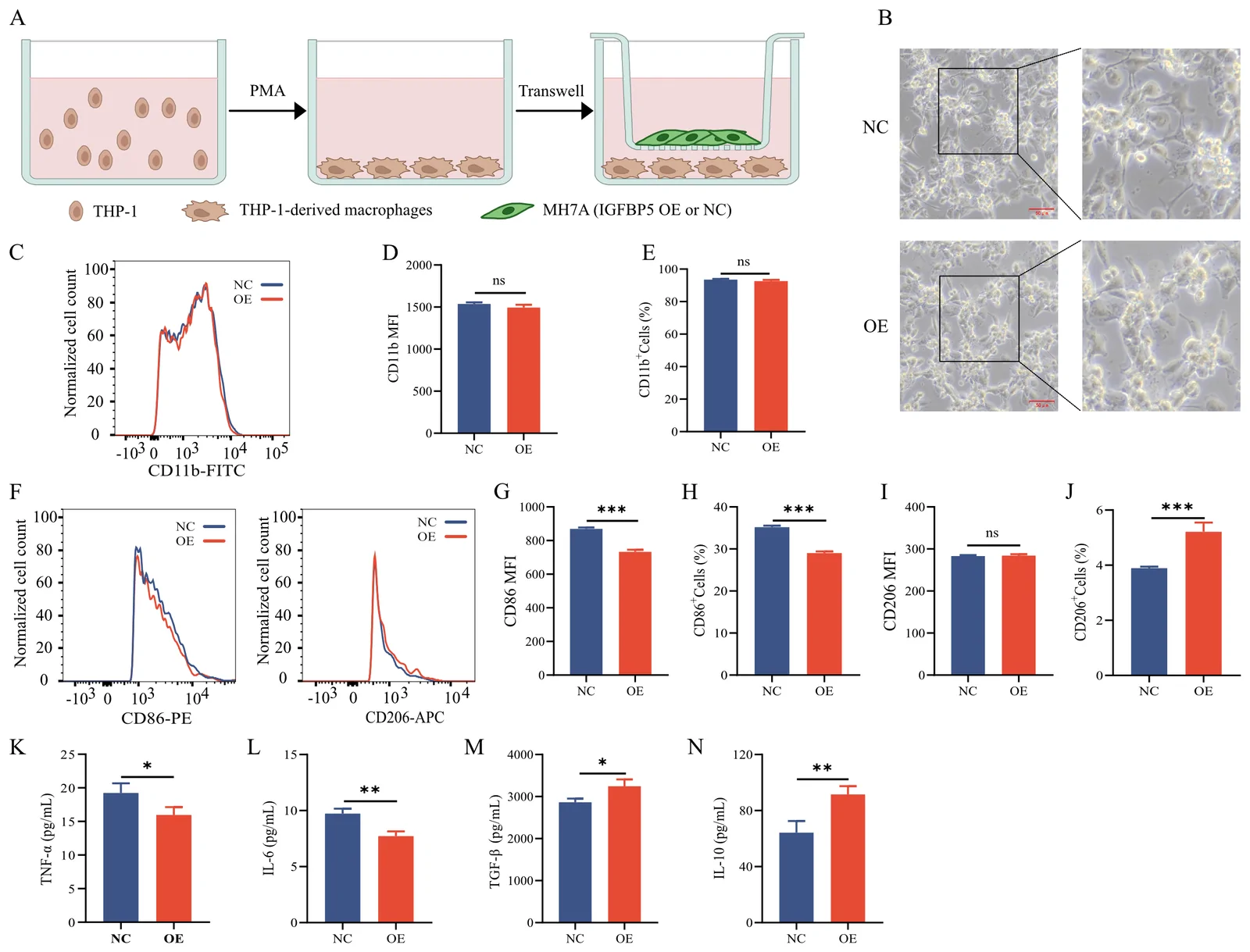
Fibroblast-derived IGFBP5 modulates macrophage phenotypic features in a coculture model. **(A)** Schematic diagram of the non-contact Transwell coculture system. THP-1-derived macrophages were cultured in the lower chamber, and control or IGFBP5-overexpressing MH7A fibroblasts were cultured in the upper insert. **(B)** Representative bright-field images of macrophages after coculture with control or IGFBP5-overexpressing fibroblasts. Scale bars = 50 μm. **(C–E)** CD11b analysis: representative overlaid fluorescence histogram normalized to the modal value **(C)**, mean fluorescence intensity (MFI) **(D)**, and percentage of CD11b-positive cells **(E)**. **(F–J)** CD86/CD206 analysis (n = 4). Representative overlaid fluorescence histograms are shown in **(F)**. CD86 MFI **(G)** and the percentage of CD86-positive/CD206-negative macrophages **(H)** were reduced in the IGFBP5-overexpression group. CD206 MFI was not significantly changed **(I)**, whereas the percentage of CD86-negative/CD206-positive macrophages was increased **(J)**. **(K–N)** ELISA measurement of TNF-α **(K)**, IL-6 **(L)**, TGF-β **(M)**, and IL-10 **(N)** in coculture supernatants (n = 3). Two-group comparisons were analyzed using two-tailed Student’s t-tests. *P < 0.05, **P < 0.01, ***P < 0.001; ns, not significant.

To examine whether fibroblast-derived IGFBP5 affects macrophage polarization, we established a non-contact Transwell coculture system. PMA-differentiated THP-1 macrophages were seeded in the lower chamber, and MH7A cells transduced with control vector or IGFBP5 overexpression vector were seeded in the upper insert (**Figure 7A**). After coculture, macrophages in the IGFBP5-overexpression group showed a more elongated morphology than those in the control group (**Figure 7B**).

Flow cytometry showed no significant difference in CD11b expression, including the percentage of CD11b-positive cells and mean fluorescence intensity, between the two groups (**Figures 7C–E**). In contrast, macrophages cocultured with IGFBP5-overexpressing fibroblasts showed lower CD86 mean fluorescence intensity and a lower proportion of CD86-positive/CD206-negative macrophages (**Figures 7F–H**). CD206 mean fluorescence intensity was not significantly changed, whereas the proportion of CD86-negative/CD206-positive macrophages was increased (**Figures 7I, J**). The complete macrophage gating strategy and representative CD86/CD206 contour plot are shown in **Supplementary Figure 8C**.

We next measured cytokine levels in the coculture supernatants. Compared with the control group, coculture with IGFBP5-overexpressing fibroblasts reduced TNF-α and IL-6 levels and increased TGF-β and IL-10 levels (**Figures 7K–N**). These results suggest that fibroblast-derived IGFBP5 alters macrophage phenotypic and cytokine profiles *in vitro* and is associated with reduced pro-inflammatory and increased anti-inflammatory features.

## 4 Discussion

Synovial fibroblasts are now recognized as active contributors to joint destruction and immune cell recruitment in RA, rather than as passive structural cells (28, 29). Therefore, identifying mechanisms that limit pathogenic fibroblast activation remains important for understanding disease progression. In this study, we identified IGFBP5 as a fibroblast-associated factor whose reduced expression characterizes pathogenic fibroblast states in RA. By integrating single-cell transcriptomic analysis, donor-level and external transcriptomic validation, in silico perturbation, *in vivo* assessment of disease-associated expression, and *in vitro* functional experiments, we found that reduced IGFBP5 expression in specific SF subsets was associated with aggressive fibroblast features and altered fibroblast-macrophage communication.

Our scRNA-seq analysis provided cell-type-specific information on the distribution of IGFBP5 in RA synovium. Previous bulk RNA studies have sometimes produced inconsistent results because of the cellular heterogeneity of synovial tissues (30). By analyzing the synovium at single-cell resolution, we found that IGFBP5 was not uniformly reduced across all cell populations, but was mainly decreased in inflammatory and fibrotic SF subpopulations. These subclusters expressed high levels of IL6, FAP, CDH11, and THY1, which are markers associated with synovial inflammation, invasiveness, and bone erosion (11, 31, 32). Previous studies have shown that pathways such as Notch3 signaling promote the expansion of THY1^+^ perivascular fibroblasts in arthritis (12). Recent work by Davidson et al. further highlighted that synovial fibroblast composition and spatial identity may contribute to site-specific susceptibility to inflammatory arthritis (33). Their study identified developmental differences in stromal-cell composition, including enrichment of PI16-positive fibroblasts in inflammation-prone joints, supporting the concept that fibroblast abundance and state are important determinants of the local inflammatory microenvironment. These observations further emphasize the importance of fibroblast heterogeneity when interpreting arthritis-associated stromal changes. Our data suggest that reduced IGFBP5 expression may represent an additional molecular feature associated with the emergence of pathogenic fibroblast states. In contrast, IGFBP5 expression was enriched in PRG4-positive resting and lining fibroblasts, which are linked to joint lubrication and synovial homeostasis (34). Pseudotime analysis further suggested that IGFBP5 expression decreases along the inferred transition from resting and lining fibroblast states toward inflammatory and fibrotic phenotypes.

In silico knockout analysis predicted that IGFBP5 loss was associated with reduced enrichment of “negative regulation of growth” and with increased inflammatory and tissue-remodeling signatures. Consistent with this prediction, IGFBP5 overexpression in RA synovial fibroblasts reduced proliferation, migration, and invasion, while increasing apoptosis. RA fibroblasts are known to display tumor-like features, including apoptosis resistance, which is partly related to metabolic reprogramming and dysregulated apoptotic pathways (35). Our results suggest that IGFBP5 may help restrain these aggressive fibroblast properties. Of note, IGFBP5 overexpression did not increase SA-β-gal staining or TGF-β secretion, suggesting that the reduced fibroblast activity was not accompanied by an evident senescence phenotype. This is relevant because senescent SFs can contribute to RA pathology through the secretion of pro-inflammatory senescence-associated secretory phenotype factors such as MMPs and IL-6 (36, 37). These findings suggest that IGFBP5 may reduce fibroblast activity mainly through effects on proliferation and apoptosis, rather than by inducing a senescence-like phenotype.

In addition to its effects on fibroblast behavior, our study also suggests a role for fibroblast-derived IGFBP5 in macrophage phenotypic regulation. The RA synovial microenvironment is maintained in part by reciprocal interactions between SFs and macrophages (38, 39). Activated SFs can promote macrophage activation through factors such as GM-CSF, CCL2, and IL-6, thereby contributing to inflammatory amplification (7, 40, 41). In our CellChat analysis, fibroblasts showed predicted interactions with macrophages, and this was further examined using a non-contact Transwell coculture system. Macrophages cocultured with IGFBP5-overexpressing fibroblasts showed reduced M1-like features, including lower CD86 expression and decreased TNF-α and IL-6 secretion, together with increased M2-like features, including higher CD206 expression and elevated TGF-β and IL-10 levels (42, 43). These findings suggest that fibroblast-derived IGFBP5 can influence macrophage polarization *in vitro*. Mechanistically, extracellular IGFBP5 may act by modulating local IGF-1/IGF-1R signaling (44) or by engaging cell surface receptors (45), such as integrin αVβ3 or LRP-1, which are expressed on macrophages and have been implicated in IGF-independent IGFBP5 signaling (46). Further studies are needed to define the receptor and downstream signaling pathways involved in this process.

From a translational perspective, the reduction of IGFBP5 in pathogenic RA fibroblast subsets suggests that this molecule may have potential value as a biomarker or therapeutic target. Current clinical assessment of RA relies partly on systemic inflammatory markers such as C-reactive protein and erythrocyte sedimentation rate, which do not fully reflect local synovial pathology or cellular heterogeneity (47). Because IGFBP5 is secreted by fibroblasts and reduced during fibroblast activation, measuring IGFBP5 in synovial fluid or serum may help evaluate its association with synovial inflammation or disease activity. However, this possibility requires validation in independent patient cohorts. Our *in vitro* overexpression experiments support a potential protective function of IGFBP5 at the cellular level, but therapeutic efficacy has not been established *in vivo*. Future studies should determine whether local restoration of IGFBP5, such as recombinant IGFBP5 administration or gene delivery, can reduce synovial inflammation and joint destruction. Given the context-dependent biology of IGFBP5, future translational studies should evaluate its utility specifically within RA synovial pathotypes (14).

This study has several limitations. First, NC and RA scRNA-seq samples originated from independent datasets, and residual dataset-associated heterogeneity cannot be completely excluded despite Harmony integration, donor-level pseudo-bulk analysis, and external transcriptomic validation.

Second, the CIA experiments were designed to evaluate disease-associated IGFBP5 expression rather than therapeutic efficacy. No IGFBP5 intervention or genetic loss-of-function group was included, and therefore the present study cannot establish an *in vivo* causal or therapeutic effect of IGFBP5. Additional qRT-PCR/immunoblot validation of macrophage polarization markers and *in vivo* IGFBP5 intervention would further strengthen mechanistic conclusions. Third, macrophage polarization was evaluated using a THP-1-derived coculture model. Although CD86/CD206 phenotypes and cytokine secretion were assessed, these findings require confirmation using primary macrophages and *in vivo* macrophage populations.

## 5 Conclusion

Our findings indicate that reduced IGFBP5 expression is associated with pathogenic synovial fibroblast states in RA. *In vitro* restoration of IGFBP5 attenuated fibroblast proliferation, migration, and invasion and was accompanied by reduced pro-inflammatory and increased anti-inflammatory macrophage features in a non-contact coculture model. Together, these results identify IGFBP5 as a candidate fibroblast-associated pathway involved in synovial fibroblast–macrophage interactions, while *in vivo* intervention studies will be required to establish causality and therapeutic efficacy.

## Data Availability

Publicly available datasets were analyzed in this study. This data can be found here: NCBI GEO repository, accession numbers GSE246416 and GSE200815.
