# Supplementary material for: Reduced fibroblast-derived IGFBP5 is associated with pathogenic synovial fibroblast states and altered macrophage polarization in rheumatoid arthritis
Source: Front Immunol. 2026 Aug 26;17:1873778. doi: 10.3389/fimmu.2026.1873778 (PMC13553944; doi:10.3389/fimmu.2026.1873778)
Supplement: Supplementary file 1 [file DataSheet1.pdf]

Supplementary Figures

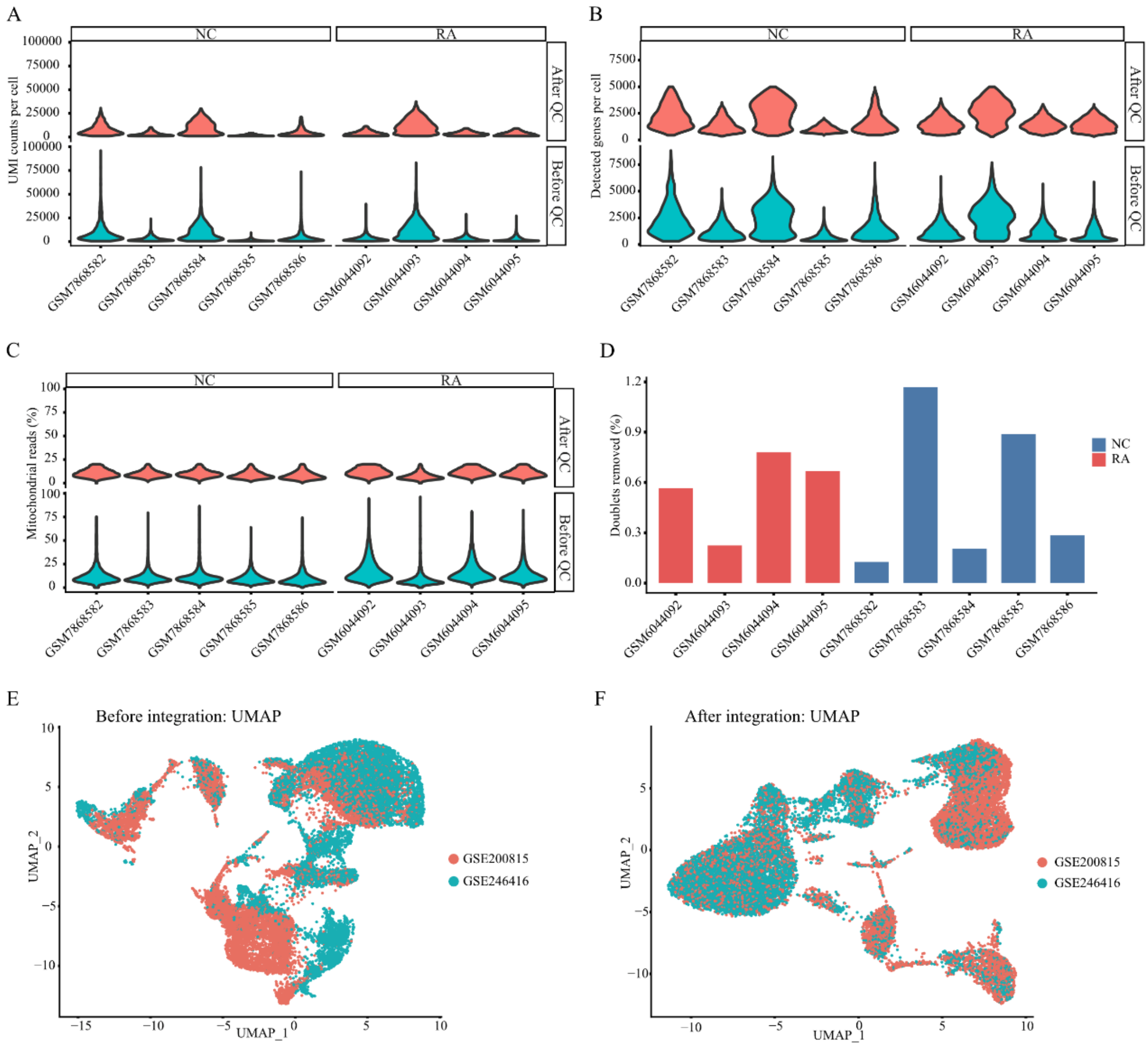

Supplementary Figure S1

Quality-control assessment and Harmony integration of the two scRNA-seq datasets. (**A-C**) Distributions of UMI counts per cell (A), detected genes per cell (B), and mitochondrial transcript proportions (C) for individual NC and RA samples before and after quality-control filtering. (**D**) Percentage of doublets removed from each sample. (**E-F**) UMAP visualizations colored by dataset origin before (E) and after (F) Harmony integration. Harmony reduced dataset-associated separation but was not assumed to eliminate all cross-dataset heterogeneity.

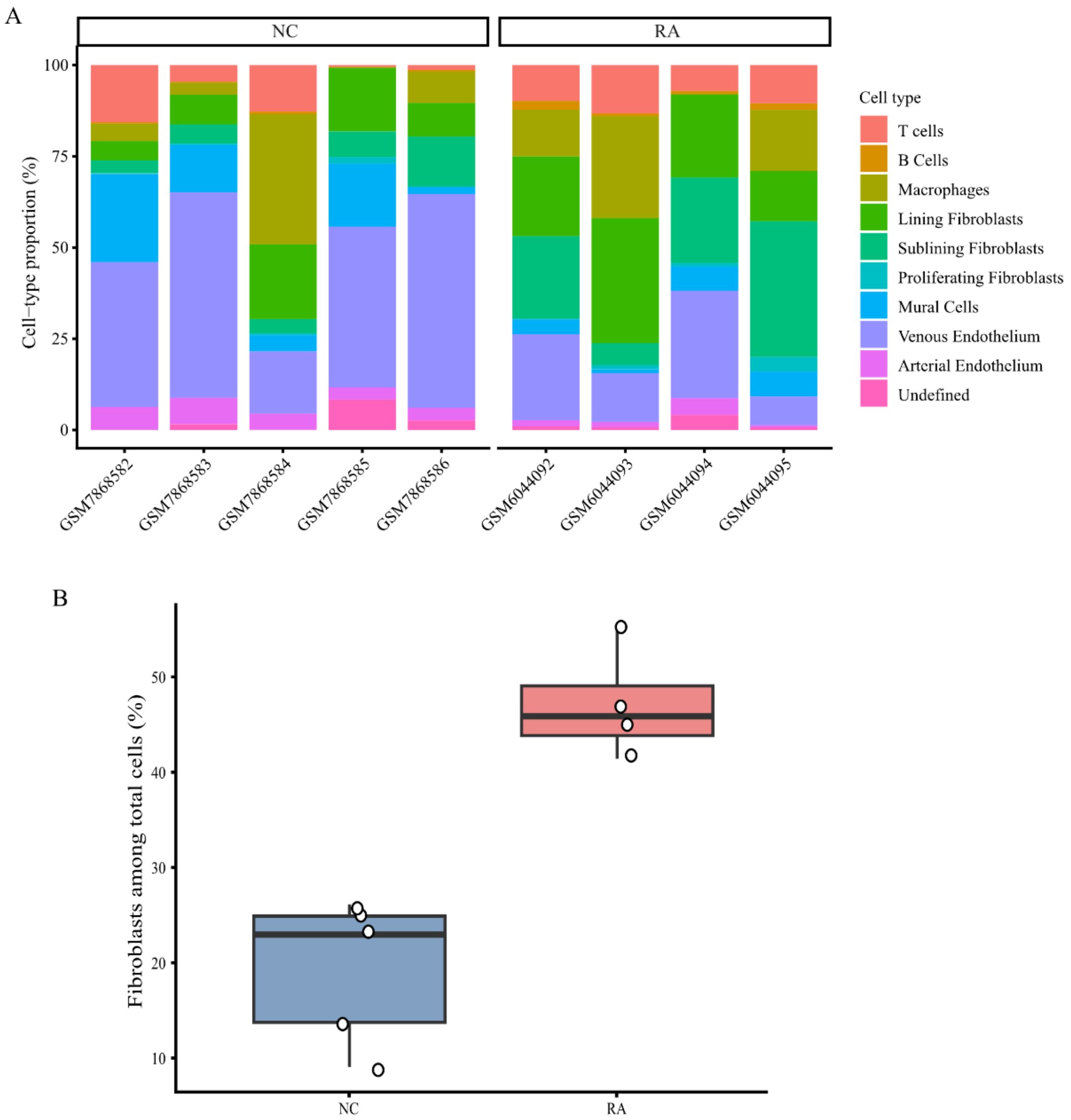

Supplementary Figure S2

Sample-level cellular composition and fibroblast abundance. (**A**) Stacked bar plots showing the relative proportions of the major annotated cell populations in each individual NC and RA sample. Each bar represents one sample. (**B**) Fibroblast abundance as a percentage of total retained cells in each sample; each point represents one sample. These plots are descriptive and show inter-sample and cross-dataset variation in cellular composition.

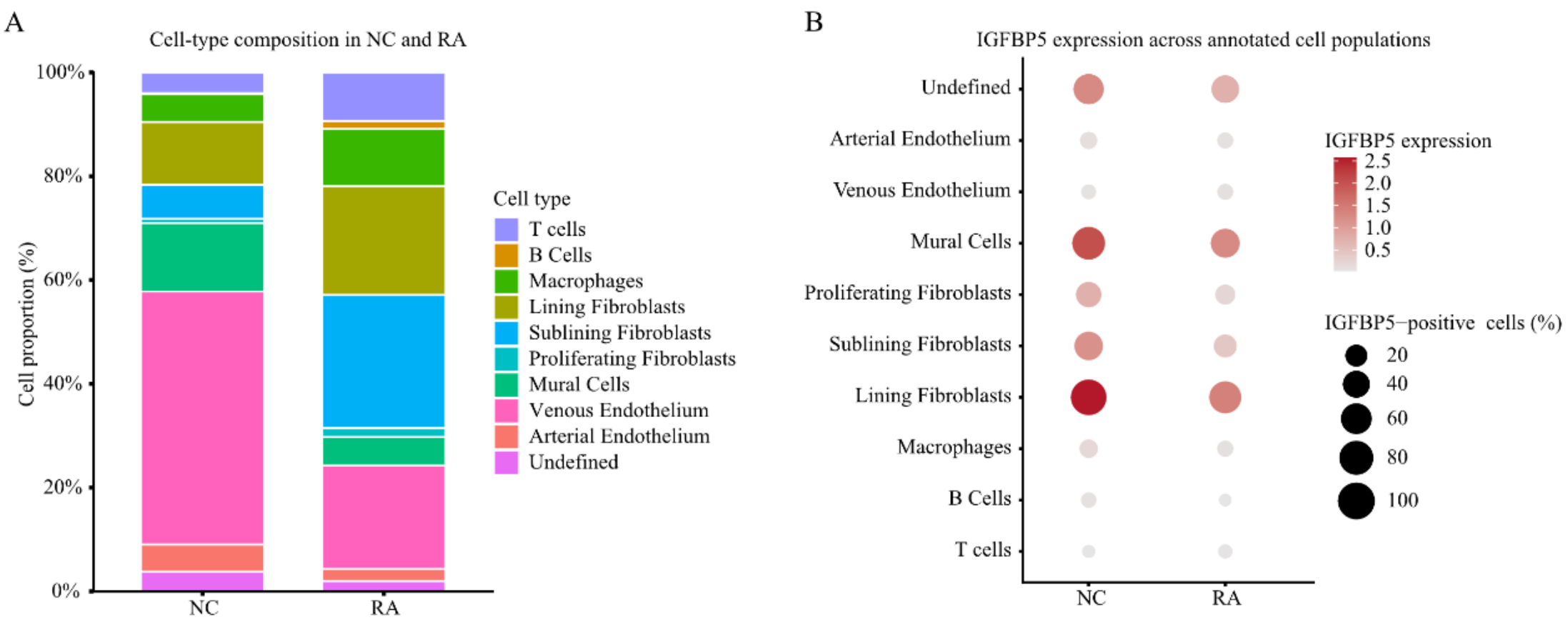

Supplementary Figure S3

Pooled NC-versus-RA cell-type composition and IGFBP5 expression across annotated cell populations. (**A**) Pooled relative proportions of annotated cell populations in NC and RA. (**B**) Dot plot summarizing IGFBP5 expression across annotated populations in NC and RA; dot size indicates the percentage of IGFBP5-positive cells, defined as cells with detectable non-zero IGFBP5 RNA counts, and color intensity indicates mean log-normalized IGFBP5 expression.

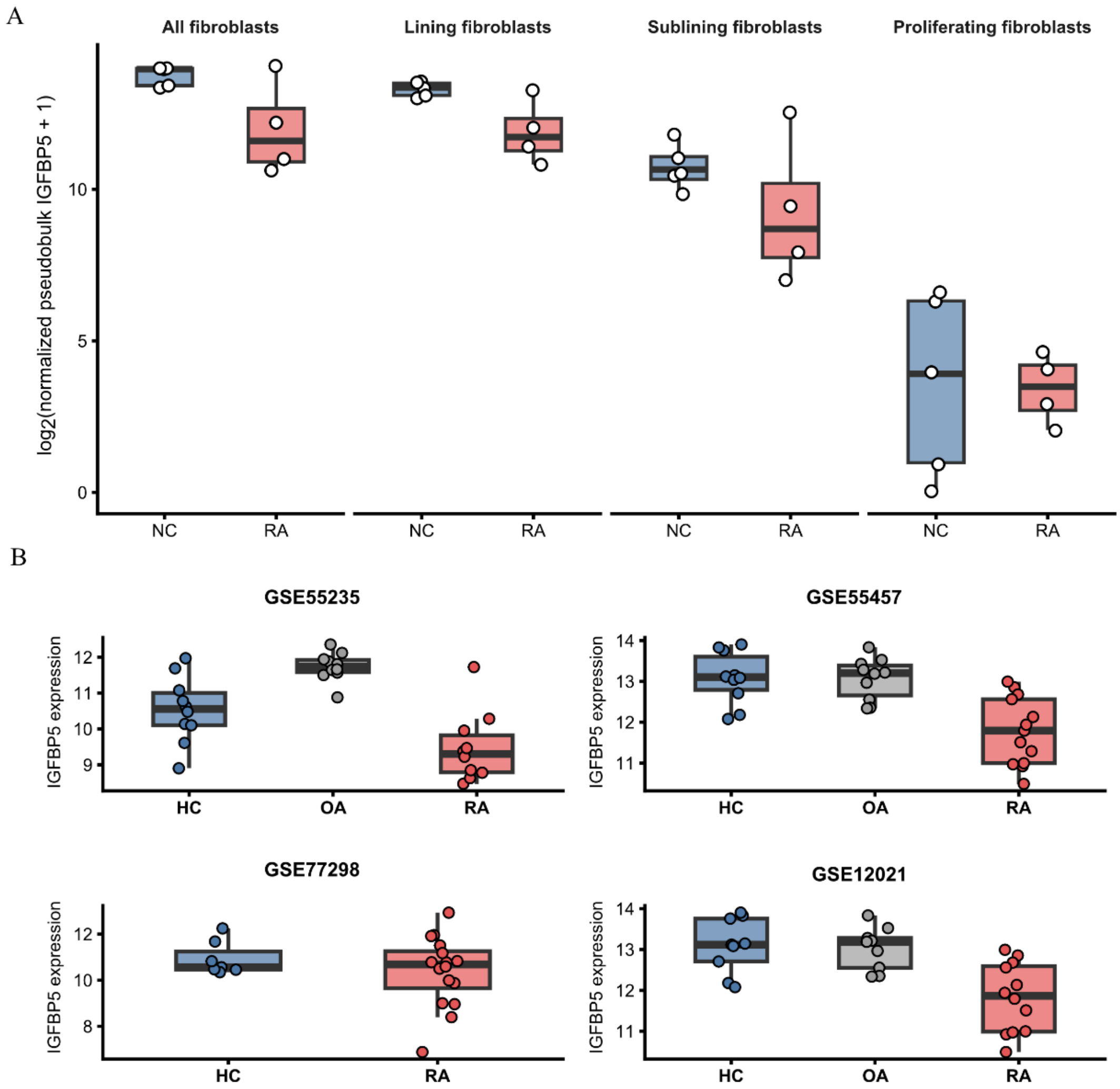

Supplementary Figure S4

Donor-level pseudo-bulk and external transcriptomic validation of IGFBP5 expression. (**A**) Donor-level pseudo-bulk IGFBP5 expression in all fibroblasts and in lining, sublining, and proliferating fibroblasts. Each point represents one donor/sample; differential expression was assessed using DESeq2. (**B**) IGFBP5 expression in external synovial transcriptomic datasets (GSE55235, GSE55457, GSE77298, and GSE12021-GPL96). Each point represents one sample. Each dataset was analyzed independently using limma without cross-dataset merging. HC, healthy control; OA, osteoarthritis.

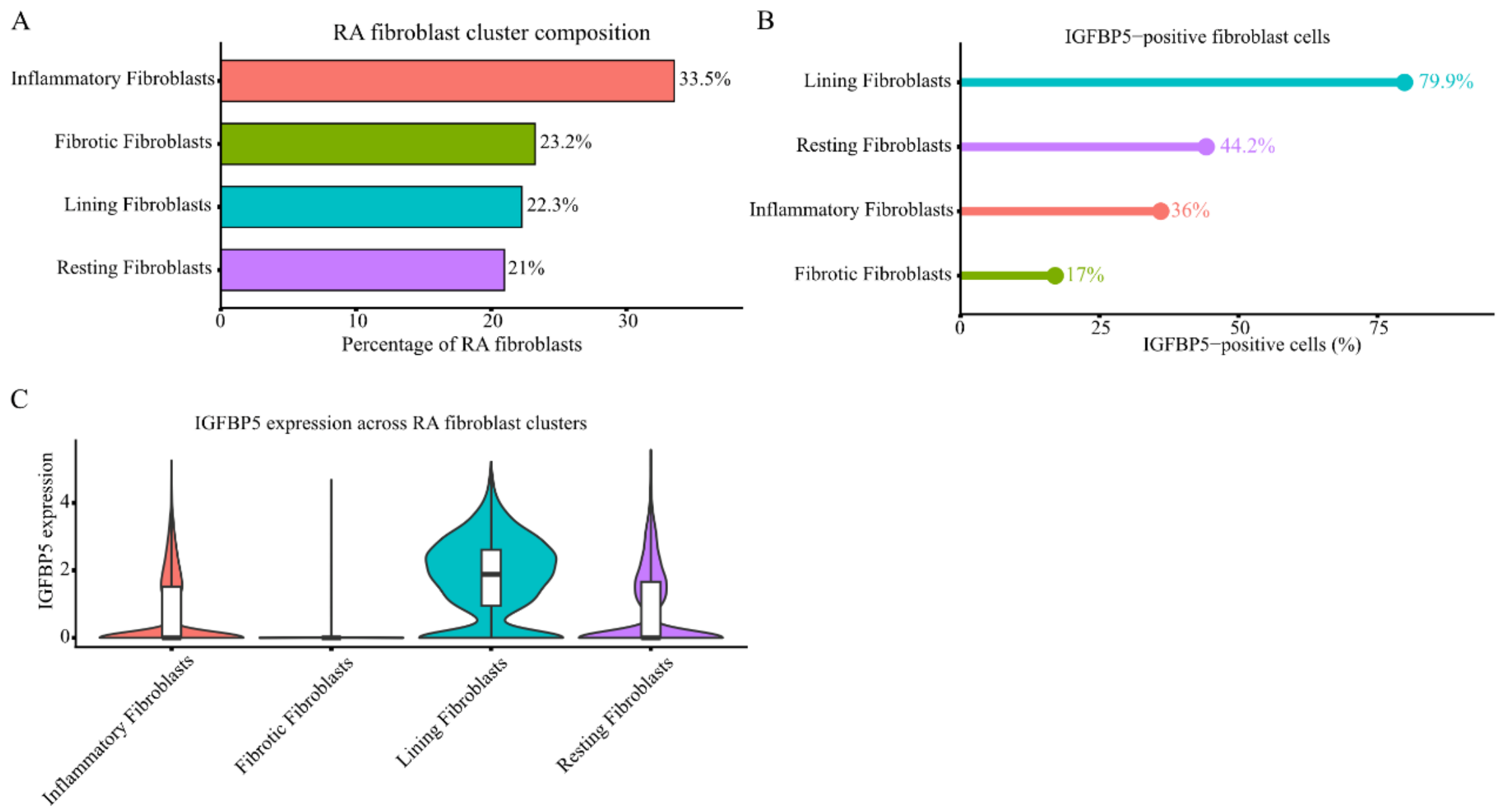

Supplementary Figure S5

Distribution of RA fibroblast subsets and IGFBP5 expression. (**A**) Relative proportions of inflammatory, fibrotic, lining, and resting fibroblasts within the RA fibroblast compartment. (**B**) Percentage of IGFBP5-positive cells within each RA fibroblast subset; IGFBP5-positive cells were defined as cells with detectable non-zero IGFBP5 RNA counts. (**C**) Distribution of log-normalized IGFBP5 expression across the four RA fibroblast subsets.

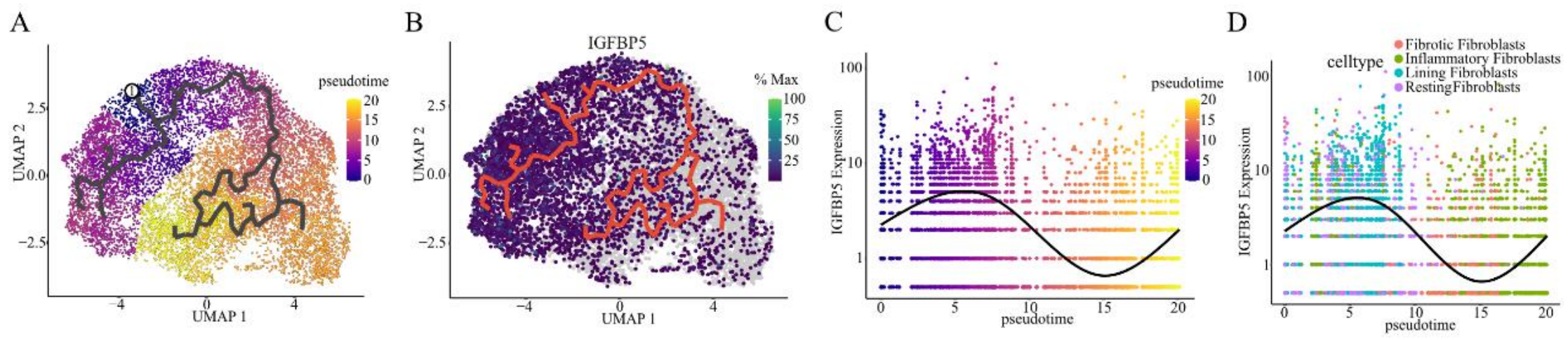

Supplementary Figure S6

Pseudotime analysis of RA fibroblast states. (**A**) Pseudotime trajectory of RA synovial fibroblasts overlaid on the UMAP projection. The root of the trajectory (node 1) is assigned to the Resting/Lining clusters, progressing towards the Inflammatory and Fibrotic subsets (dark blue to yellow color scale indicates pseudotime progression). (**B**) Expression levels of IGFBP5 projected onto the inferred developmental trajectory, highlighting its enrichment near the root. (**C**) Scatter plot showing the global expression dynamics of IGFBP5 as a function of pseudotime. The black curve represents the fitted trend, demonstrating a continuous decline in IGFBP5 expression as cells transition along the trajectory. (**D**) IGFBP5 expression along pseudotime, with individual cells colored by their respective fibroblast subpopulation identities. The plot illustrates that early-pseudotime cells (Resting and Lining Fibroblasts) express higher levels of IGFBP5, which is progressively lost as they differentiate into late-pseudotime pathogenic states (Inflammatory and Fibrotic Fibroblasts).

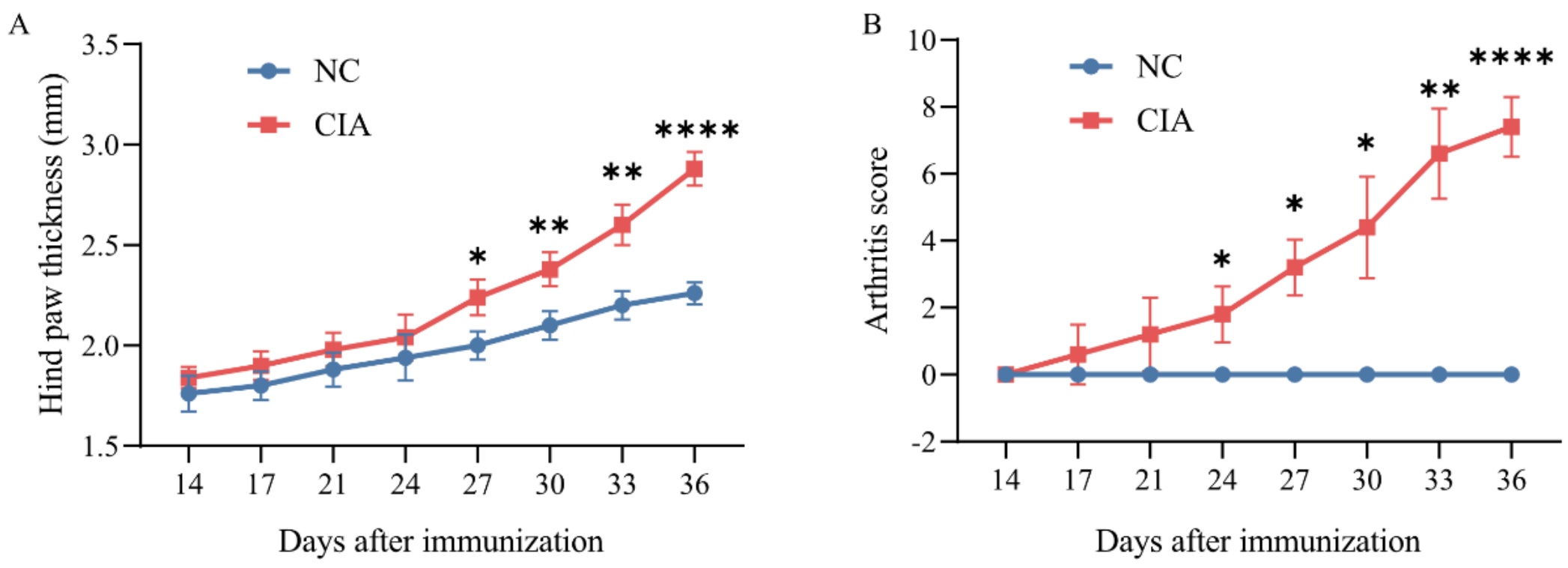

Supplementary Figure S7

Longitudinal clinical assessment of the CIA model. (**A**) Hind-paw thickness and (**B**) clinical arthritis score measured on days 14, 17, 21, 24, 27, 30, 33, and 36 after the initial immunization in NC and CIA mice (n = 5 mice per group). Data are presented as mean ± SD. Longitudinal group differences were analyzed using two-way repeated-measures ANOVA with Geisser-Greenhouse correction followed by Sidak-adjusted multiple comparisons.

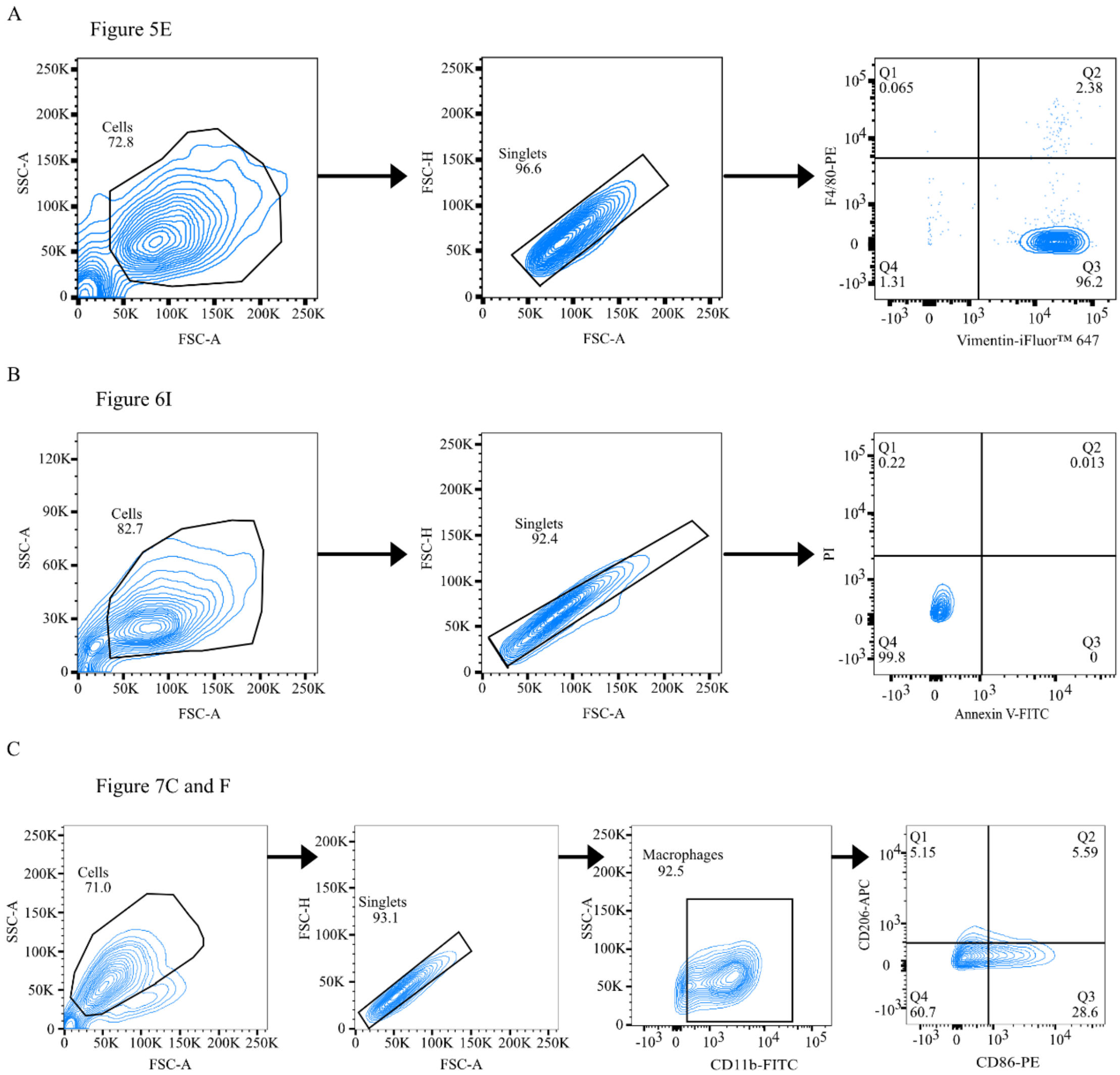

Supplementary Figure S8

Flow-cytometry gating strategies. (**A**) Gating strategy for primary fibroblast purity analysis corresponding to Figure 5E: cells were gated on FSC-A/SSC-A, singlets were selected using FSC-A/FSC-H, and Vimentin/F4/80 expression was then assessed. (**B**) Gating strategy for Annexin V-FITC/PI apoptosis analysis corresponding to Figure 6I after cell and singlet selection. (**C**) Gating strategy for macrophage analysis corresponding to Figure 7C and 7F-J: cells were selected on FSC-A/SSC-A, singlets were gated using FSC-A/FSC-H, CD11b-positive macrophages were identified, and CD86/CD206 expression was evaluated using a contour plot with identical quadrant boundaries for compared groups.

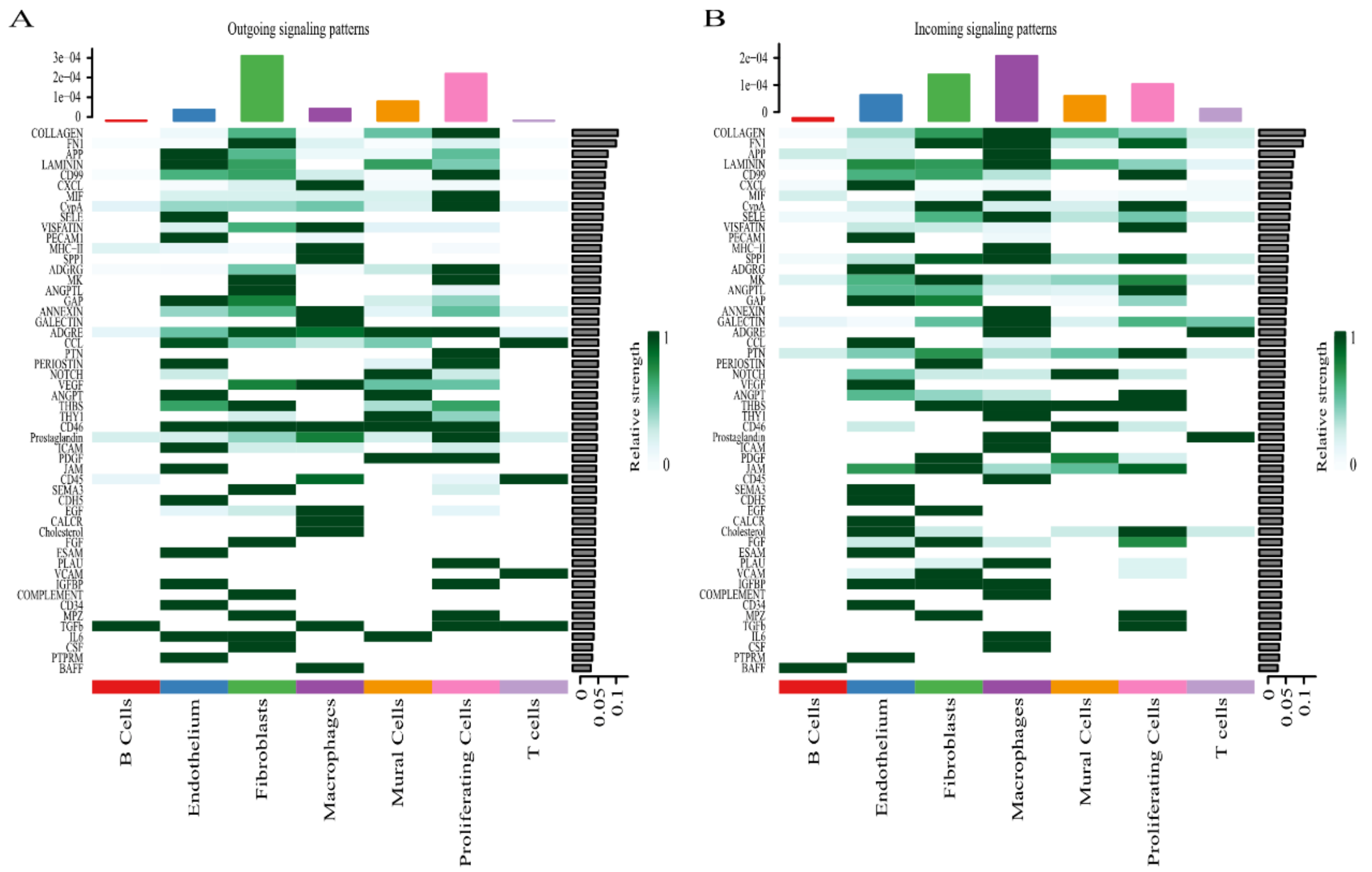

Supplementary Figure S9

CellChat signaling-pattern analysis in RA synovium. CellChat was performed using pooled cells from the RA-only GSE200815 dataset (n = 4 donors; N = 27,699 cells). (**A**) Outgoing signaling patterns and (**B**) incoming signaling patterns across the seven analyzed cell populations.

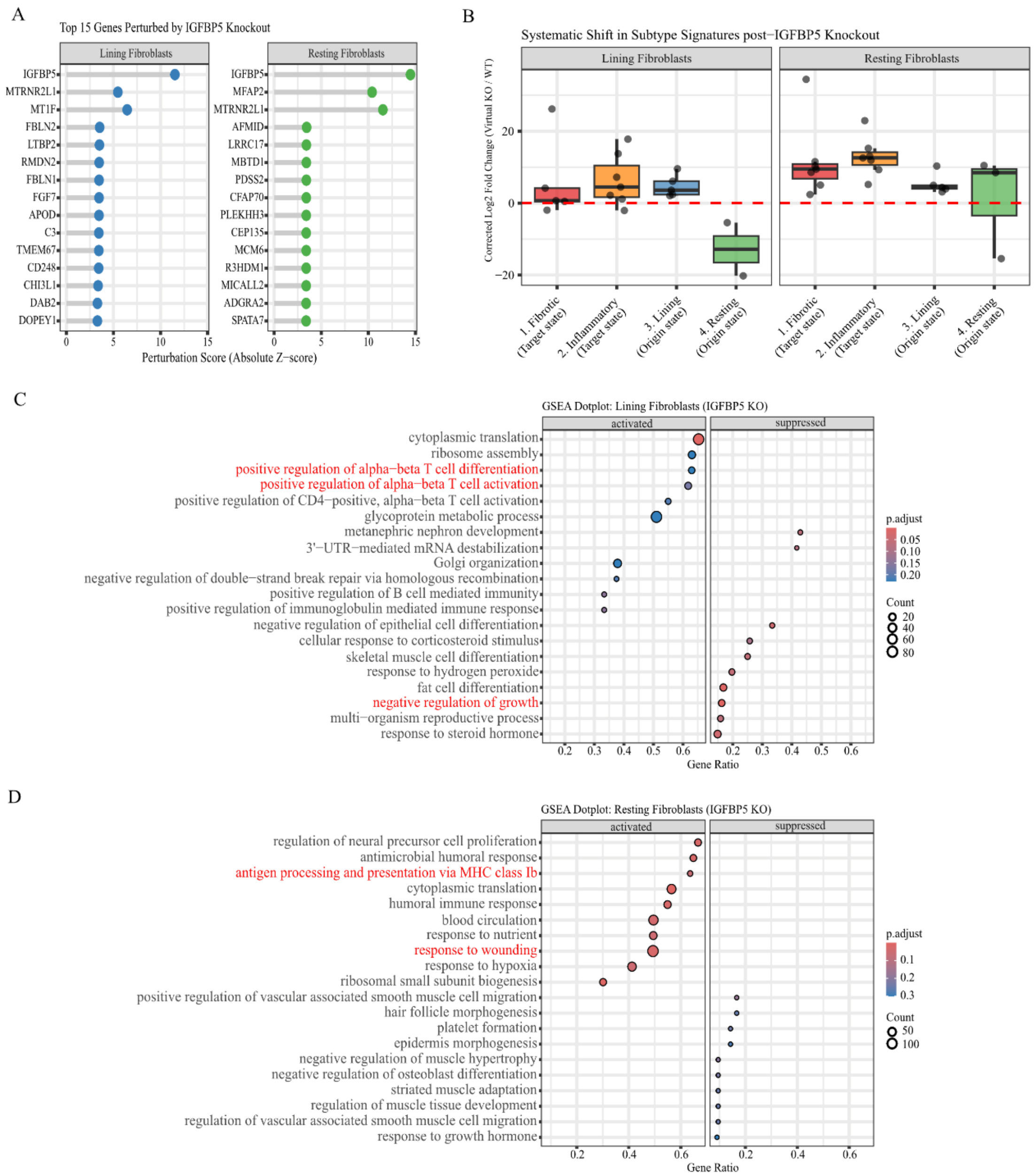

Supplementary Figure S10

In silico IGFBP5 perturbation and pathway-enrichment analysis. (**A**) Top 15 perturbed genes resulting from the virtual knockout (KO) of IGFBP5 in Lining and Resting Fibroblast clusters, ranked by absolute Z-score. (**B**) Systematic shift analysis demonstrating that IGFBP5 KO functionally reprograms homeostatic fibroblasts (Lining and Resting origin states), driving their transcriptomic signatures closely toward the pathogenic Inflammatory and Fibrotic target states. (**C-D**) GSEA dot plots illustrating the significantly activated and suppressed biological processes following IGFBP5 KO in (C) Lining Fibroblasts and (D) Resting Fibroblasts. Note the prominent suppression of the negative regulation of growth alongside the activation of immune responses and wound-healing pathways.

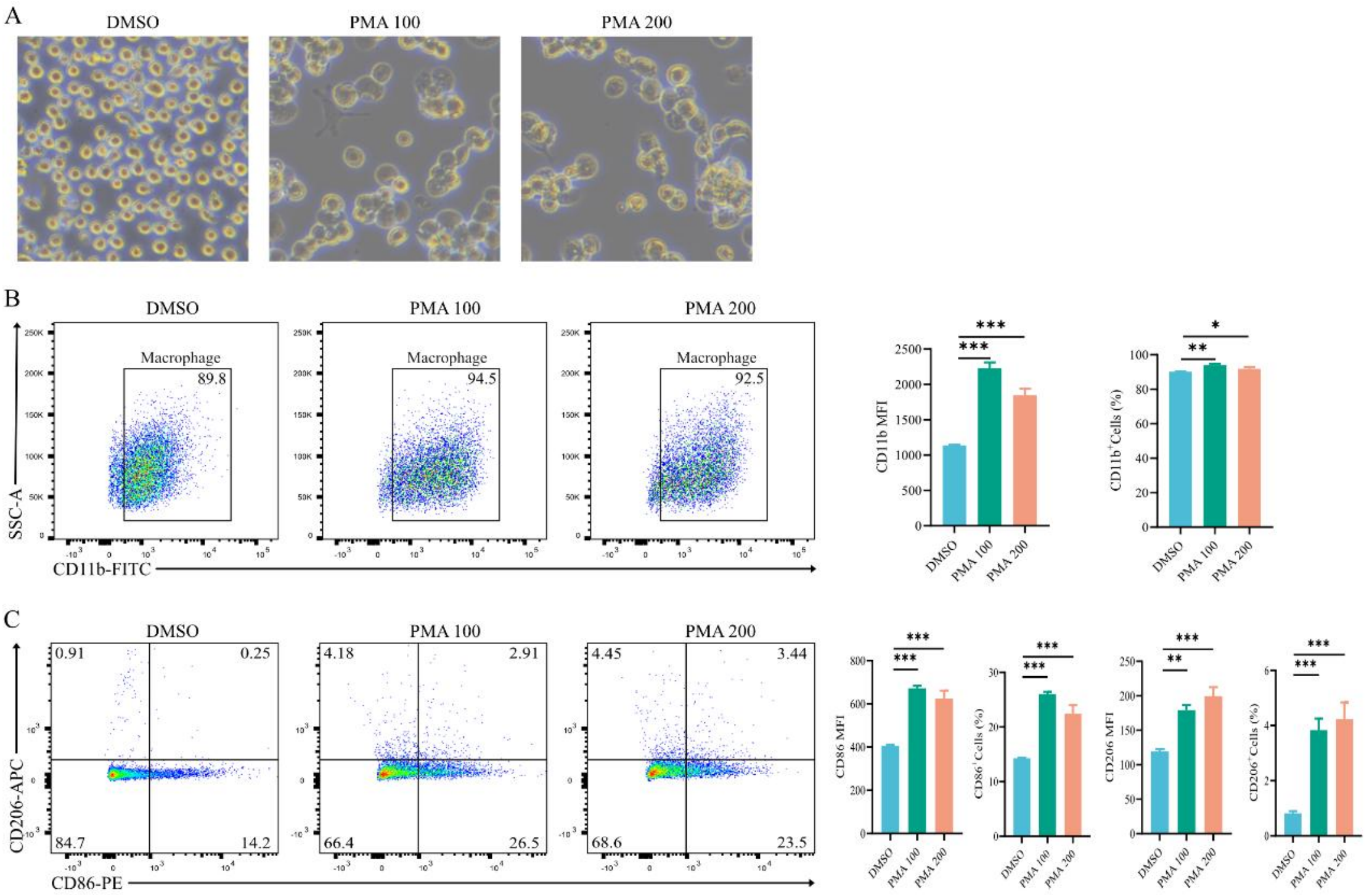

Supplementary Figure S11

Optimization of PMA-induced THP-1 macrophage differentiation. THP-1 cells were treated with DMSO, 100 ng/mL PMA, or 200 ng/mL PMA. (**A**) Representative bright-field morphology. (**B**) Representative CD11b flow-cytometry plots and corresponding quantification (n = 3). (**C**) Representative CD86/CD206 quadrant plots and corresponding quantitative summaries (n = 3). Where indicated, group comparisons were analyzed using one-way ANOVA followed by Tukey's multiple-comparison test. *P < 0.05, **P < 0.01, ***P < 0.001, ****P < 0.0001; ns, not significant.
